# Estradiol Ameliorates Postmenopausal Bladder Dysfunction by Restoring Mitophagy via the miRNA-200/KLF4/mTOR Signaling Pathway

**DOI:** 10.3390/ijms27177529

**Published:** 2026-08-22

**Authors:** Kuang-Shun Chueh, Jian-He Lu, Jing-Wen Mao, Bin-Nan Wu, Cheng-Yu Long, Zhi-Feng Miao, Tai-Jui Juan, Rong-Jyh Lin, Shu-Mien Chuang, Mei-Chen Shen, Ting-Wei Sun, Mei-Chin Lu, Yung-Shun Juan

**Affiliations:** 1 Graduate Institute of Clinical Medicine, College of Medicine, Kaohsiung Medical University, Kaohsiung 80708, Taiwan; spacejason69@yahoo.com.tw (K.-S.C.); urolong@yahoo.com.tw (C.-Y.L.); rjlin@kmu.edu.tw (R.-J.L.); 2 Department of Urology, Kaohsiung Medical University Hospital, Kaohsiung 80756, Taiwan; blast2337@gmail.com (J.-W.M.); u9181002@gmail.com (S.-M.C.); bear5824@gmail.com (M.-C.S.);; 3 Department of Urology, College of Medicine, Kaohsiung Medical University, Kaohsiung 80708, Taiwan; 4 Emerging Compounds Research Center, Department of Environmental Science and Engineering, College of Engineering, National Pingtung University of Science and Technology, Pingtung 91201, Taiwan; toddherpuma@yahoo.com.tw; 5 Department of Pharmacology, Graduate Institute of Medicine, College of Medicine, Kaohsiung Medical University, Kaohsiung 80708, Taiwan; binnan@kmu.edu.tw; 6 Department of Obstetrics and Gynecology, Kaohsiung Medical University Hospital, Kaohsiung 80708, Taiwan; 7 Department of Obstetrics and Gynecology, Kaohsiung Municipal Hsiao-Kang Hospital, Kaohsiung 80708, Taiwan; 8 Regenerative Medicine and Cell Therapy Research Center, Kaohsiung Medical University, Kaohsiung 80708, Taiwan; 9 Department of Surgery, Division of Colorectal Surgery, Kaohsiung Medical University Hospital, Kaohsiung Medical University, Kaohsiung 80708, Taiwan; zfmiao@yahoo.com; 10 Department of Surgery, Division of Urological Surgery, Kaohsiung Armed Forces General Hospital, Kaohsiung 80284, Taiwan; terryjuan@gmail.com; 11 Department of Parasitology, School of Medicine, College of Medicine, Kaohsiung Medical University, Kaohsiung 80708, Taiwan; 12 Graduate Institute of Marine Biology, National Dong Hwa University, Pingtung 944, Taiwan; 13 National Museum of Marine Biology and Aquarium, Pingtung 944, Taiwan

**Keywords:** ovarian hormone deficiency, overactive bladder, estradiol, mitophagy, miRNA-200, Krüppel-like factor 4

## Abstract

Postmenopausal ovarian hormone deficiency (OHD) contributes to overactive bladder (OAB) through oxidative stress, mitochondrial dysfunction, and dysregulated estrogen receptor (ER) signaling. This study investigated whether estradiol (E2) alleviates dysfunction by modulating the ER/Smad/miRNA-200/KLF4/mTOR signaling pathway to restore mitochondrial quality control. Thirty female Sprague-Dawley rats were initially allocated to Sham, Ovariectomy (OVX) and OVX + E2 group; after attrition during the 12-month protocol, six surviving animals per group were included in the principal analyses. The OVX + E2 group received daily intramuscular E2 (IM, 30 μg/kg/day) for one month. Bladder function was assessed via micturition volume and frequency by metabolic cages, cystometrograms, and contractility assays. Mechanisms were analyzed using immunofluorescence, Western blotting, transmission electron microscopy (TEM), and miRNA sequencing. OVX rats exhibited significant overactivity and compromised contractility, accompanied by upregulated ERα and downregulated ERβ/GPER. Molecularly, OHD was associated with the upregulation of the miRNA-200 family (miRNA-200a-3p, miRNA-200b-3p and miRNA-200b-5p), which was accompanied by reduced levels of KLF4 and autophagy proteins (ATG7, ATG12 and Beclin-1), as well as elevated p-mTOR expression. TEM revealed the accumulation of damaged mitochondria with ultrastructural features associated with impaired mitophagy. However, E2 treatment ameliorated these abnormalities. These improvements were associated with the restoration of TGF-β/Smad signaling, downregulation of selected miRNA-200 family members, recovery of KLF4 level, and increased expression of autophagy-related markers, accompanied by improved mitochondrial ultrastructural integrity. This cellular restoration might be correlated with improved urodynamic parameters. E2 might exert therapeutic effects by improving mitochondrial quality, potentially via the ER/Smad/miRNA-200/KLF4 signaling pathway. These findings provided mechanistic insights into estrogen-mediated protection and highlight this proposed signaling pathway as a therapeutic target for postmenopausal OAB.

## 1. Introduction

Lower urinary tract symptoms (LUTS) including urinary frequency, urgency, nocturia, and overactive bladder (OAB), are common in postmenopausal women [1,2]. Ovarian hormone deficiency (OHD) in these women has been shown to contribute to the development of OAB, which is characterized by urinary urgency, frequency and nocturia. Studies by Christmas and Russo et al. have demonstrated that estradiol (E2) treatment in postmenopausal women with vulvovaginal atrophy could improve urodynamic parameters, such as increasing maximal bladder capacity, decreasing non-voiding contractions, and alleviating urinary frequency [3,4]. Additionally, animal studies using a bilateral ovariectomy (OVX) rat model have demonstrated that estrogen depletion could induce bladder dysfunction, including decreased compliance, urothelial thinning, and increased oxidative stress [5,6,7,8]. A previous study also found that bilateral OVX-induced OHD in rats resulted in reduced bladder compliance, elevated oxidative stress, interstitial fibrosis, and increased apoptosis in the bladder mucosa [8]. Research has indicated that estrogen deficiency could damage the bladder tissue structure, while estrogen supplementation could increase bladder weight and vascular density [9]. E2 treatment has been reported to reverse these changes by enhancing angiogenesis and promoting smooth muscle remodeling [10,11]. Estrogen played a role in maintaining the bladder function, including muscle tone, blood flow, and urothelial structure. Moreover, the elasticity of urethral and bladder tissues, which affected various storage phase symptoms, was linked to estrogen levels [12].

Estrogen deficiency during menopause triggers OAB symptoms and suppresses autophagic protein expression and mitophagic clearance, leading to the accumulation of damaged mitochondria and excessive oxidative stress [13]. Specifically, mitophagy the selective degradation of dysfunctional mitochondria is critical for maintaining urothelial bioenergetics and reducing reactive oxygen species (ROS). Since autophagy is a crucial process for maintaining cellular homeostasis, its disruption by OHD contributes to bladder dysfunction. Consistent with this, a previous study demonstrated that the mTOR inhibitor rapamycin attenuated bladder overcapacity and smooth muscle hypertrophy, while also improving pathological residual urine volumes during long-term bladder outlet obstruction [14]. Similarly, in the cyclophosphamide-treated rats, bladder micturition function was significantly improved after treated with rapamycin, an autophagy inducer, which also lessened oxidative stress [15]. In a rat model of ketamine-induced cystitis, rapamycin treatment significantly increased the expression of autophagy-related proteins [16]. Although estrogen played a crucial regulatory role in the crosstalk between estrogen signaling and autophagic activity in the progression of OAB, the upstream regulatory mechanisms that govern autophagic activity in the bladder, especially under conditions of OHD, remain incompletely understood.

Estrogen signaling also exerts a significant influence on autophagy within bladder tissue, particularly under conditions of OHD. In OVX animal models, elevated expression of transforming growth factor-beta (TGF-β), as well as its downstream mediators Smad2 and Smad3, has been linked to renal fibrosis [17]. A previous study suggested that ERα could enhance autophagic activity through ROS production and activation of the MEK/ERK pathway [18]. Moreover, estrogen has been reported to counteract TGF-β-induced Smad3 activation, a pathway implicated in fibrotic progression and autophagic activity inhibition [19,20]. Therefore, estrogen signaling has been shown to influence cell survival, inflammation, angiogenesis, and epithelial repair. Estrogen receptors, acting through both genomic and non-genomic mechanisms, have been implicated in the modulation of oxidative balance, fibrotic signaling, and cellular remodeling processes.

MiRNAs regulate gene expression at the post-transcriptional level by targeting the 3′-UTR of mRNAs for degradation or translation suppression [21]. Due to their stability, tissue specificity, and detectability, circulating miRNAs have emerged as promising biomarkers for organ damage and pathological progression [21,22,23]. Increasing evidence highlights the critical role of miRNAs in urinary tract pathophysiology, including inflammation, fibrosis, and epithelial injury. In patients with OAB, altered circulating levels of multiple miRNAs have been identified, including elevated miRNA-let-7b-5p, miRNA-92a-3p, and miRNA-200c-3p [24,25]. Among these, the miRNA-200 family (miRNA-200a, miRNA-200b, miRNA-200c, miRNA-141, and miRNA-429) has attracted particular attention due to its regulatory functions in oxidative stress and autophagy. Experimental studies show that miRNA-200a directly targets ATG7 to reduce autophagic activity [26], while the downregulation of miRNA-200b under cellular stress increases ATG12 expression, facilitating autophagosome formation [27]. Additionally, TGF-β signaling suppresses the expression of miRNA-200 members [28,29], further linking them to stress responses and tissue remodeling. Krüppel-like factor 4 (KLF4), a transcription factor implicated in cellular and mitochondrial homeostasis, has been identified as a predicted and experimentally supported target of miR-200b in non-bladder cellular systems [30,31]. However, whether this regulatory relationship occurs in bladder tissue under chronic ovarian hormone deficiency remains unknown. Although estrogen deficiency, bladder remodeling, and autophagy-related signaling have each been investigated separately, it remains unclear whether estrogen receptor subtype alterations are linked to miRNA-mediated regulation of mitochondrial quality control in the chronically estrogen-deficient bladder. The present study therefore examined whether coordinated changes in ER/Smad/miR-200/KLF4/mTOR signaling are associated with mitochondrial abnormalities and bladder dysfunction in a long-term OVX model and whether these changes are reversible with E2 treatment. However, whether E2 restores bladder function by modulating the ER/Smad/miR-200/KLF4 signaling pathway to enhance mitophagy remains unclear.

The molecular mechanism underlying the cytoprotective effect of E2 treatment may involve the regulation of autophagic formation. In the present study, we hypothesized that E2 enhances autophagic activity by modulating the miRNA-200/KLF4 signaling pathway through estrogen receptor signaling. Specifically, our aim was to elucidate whether E2 mitigates OAB symptoms in OHD-induced rats by regulating the ER/Smad/miRNA-200/KLF4/mTOR pathway, thereby restoring cellular homeostasis.

## 2. Results

### 2.1. Serum Parameters After OVX

Serum E2 concentrations were presented in Table 1. In comparison with the sham group (27.05 ± 6.07 pg/mL), the serum E2 levels were significantly decreased to 7.53 ± 2.35 pg/mL in the OVX group, and to 7.08 ± 2.56 pg/mL in the OVX + E2 group before E2 treatment (both *p* < 0.001). These data indicated that bilateral OVX surgery induced E2 deficiency. After OVX surgery for 12 months, the serum E2 levels in the OVX group (7.20 ± 1.79 pg/mL) was significantly lower compared to the sham group (30.94 ± 3.34 pg/mL, *p* < 0.001). However, there was no difference between the sham group and the OVX + E2 group (28.49 ± 4.28 pg/mL, *p* = 0.222).

### 2.2. Physical Characteristics

Table 1 presents the physical indicators following 12 months of bilateral OVX. There were no significant differences among the three groups in water intake, urine output, and absolute bladder weight. Body weight was significantly increased in the OVX group compared with the Sham group and was reduced following E2 treatment. Consequently, the bladder-to-body-weight ratio was lower in the OVX group (*p* < 0.001) and increased following E2 treatment (*p* = 0.008). Because this ratio is directly influenced by body weight, absolute bladder weight was further analyzed using ANCOVA, with body weight included as a covariate. After adjustment for body weight, the overall group effect on absolute bladder weight was not statistically significant (*p* = 0.156). Therefore, the differences in the bladder-to-body-weight ratio were interpreted primarily as reflecting changes in body weight rather than alterations in absolute bladder mass. The bladder-to-body-weight ratio was therefore retained only as a descriptive parameter and was not used as evidence of pathological changes in bladder mass.

### 2.3. Estradiol Treatment Ameliorated Bladder Overactivity

**Figure 1:**
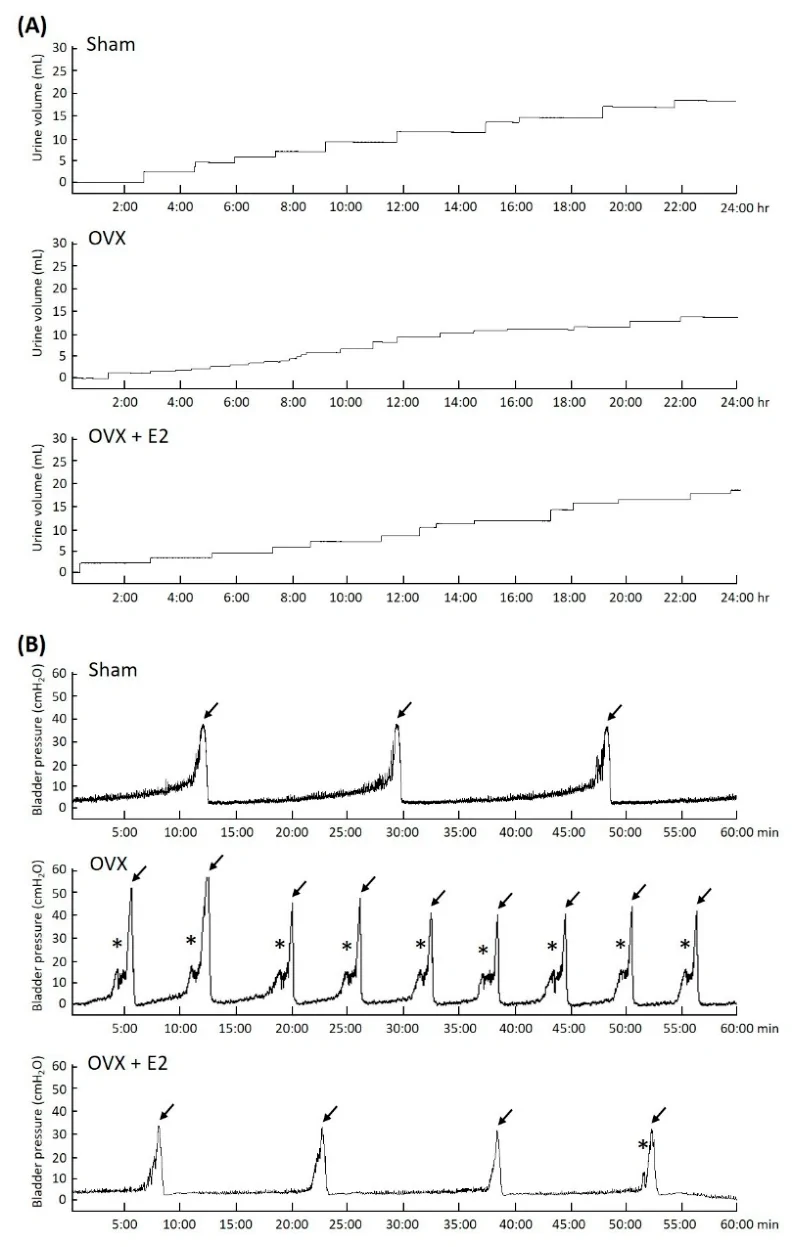
E2 supplement improved micturition behavior and ameliorated bladder detrusor overactivity in a rat model of OHD-induced OAB-like storage phenotype. (**A**) Tracing analysis of 24 h micturition behavior using a metabolic cage showed that the OVX group exhibited significant increases in bladder voiding volume and micturition frequency. (**B**) Cystometrogram recordings display micturition pressure, volume, and frequency over a 60 min period, including voiding contractions (arrows) and non-voiding contractions (asterisks). These changes were notably attenuated in the OVX + E2 group following E2 treatment. Note: E2, estradiol; OAB, overactive bladder; OHD, ovarian hormone deficiency; OVX, bilateral ovariectomy.

Bladder function was evaluated using urodynamic parameters by micturition behavior (Figure 1A) and cystometrogram (CMG) (Figure 1B). From the analysis of micturition behavior (Figure 1A and Table 1), the OVX group exhibited a higher micturition frequency and lower voided volume compared to the sham group (*p* < 0.001). In contrast, the OVX + E2 group exhibited significantly lower micturition frequency and higher voided volumes compared to the OVX group (*p* < 0.001). Therefore, E2 contributed to improved micturition behavior and alleviation of bladder overactivity.

CMG data (Figure 1B and Table 1) exhibited that the sham group depicted a regular and stable micturition pattern. In contrast, the OVX group exhibited bladder overactivity, evidenced by an increased contraction frequency (arrows), an elevated peak micturition pressure, as well as a higher number, non-voiding contractions (asterisk) between micturition and a decreased calculated voided volume (*p* < 0.001 vs. the sham group). Conversely, the OVX + E2 group exhibited significant reduction in micturition frequency, lower peak micturition pressure, fewer non-voiding contractions and increased voided volume, as compared to the OVX group (*p* < 0.001). These results suggested that the OVX-treated rats exhibited significant bladder overactivity and abnormal detrusor activity, characterized by increased micturition frequency and decreased micturition volumes. In contrast, E2 administration led to notable improvements in micturition volumes and alleviated symptoms of detrusor overactivity induced by OHD.

### 2.4. Estradiol Treatment Improved Bladder Detrusor Contractile Response

**Figure 2:**
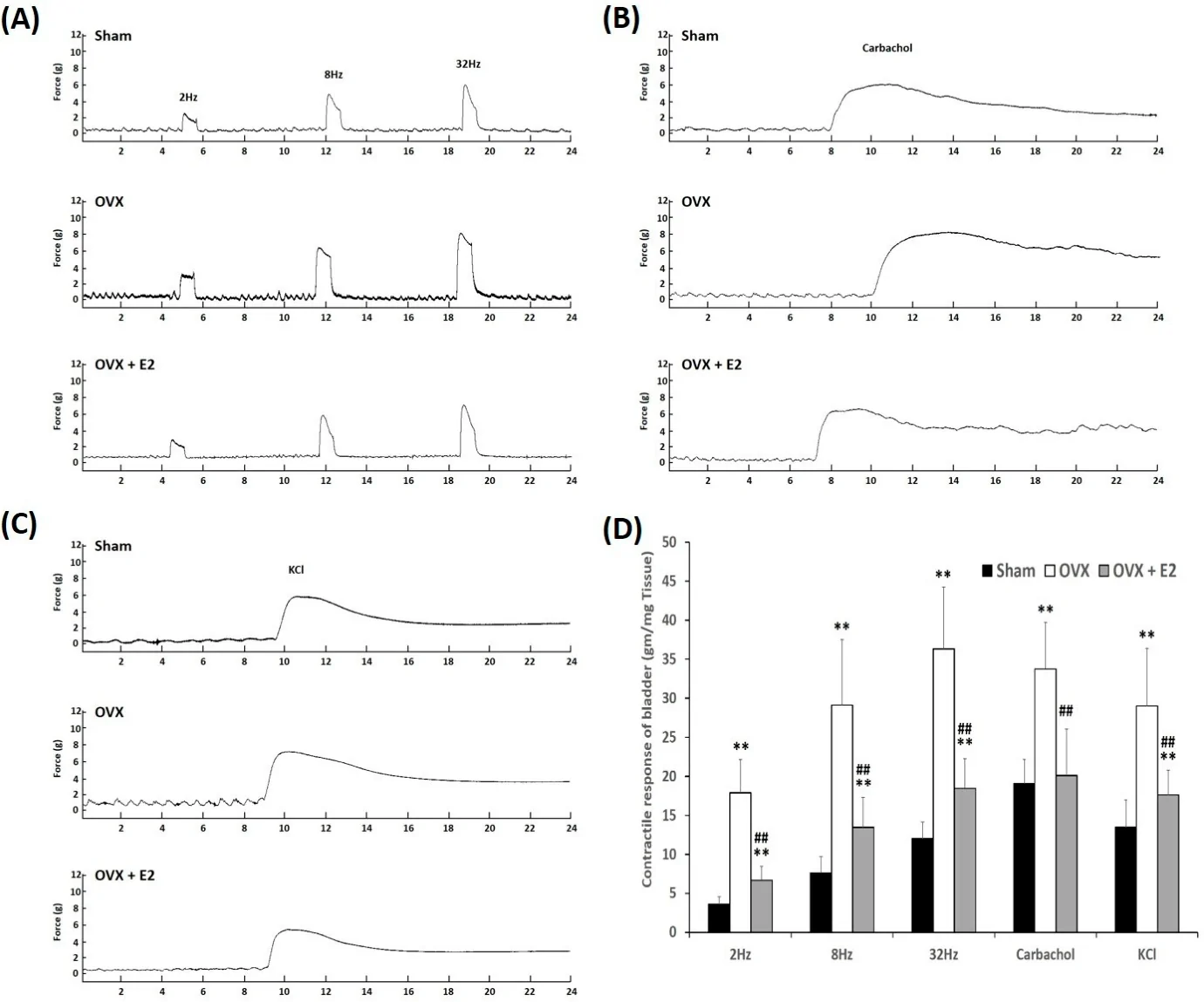
Bladder contractile responses to EFS, carbachol and KCl stimulation. After 12 months of OVX, bladder strips had higher EFS-induced contractile responses at 2, 8, and 32 Hz than the sham group. However, the OVX + E2 group had lower contractile responses compared with the OVX group (**A**,**D**). Treatment with carbachol (**B**,**D**) and KCl (**C**,**D**) in the OVX group induced higher contractile responses than the sham group, while the contractile responses were reduced in the OVX + E2 group. The E2 supplement ameliorated contractile responses by using muscle strips for synaptic transmission, receptor response, and smooth muscle contraction. Note: EFS, electrical field stimulation; OVX, bilateral ovariectomy; E2, estradiol. Data were represented as mean ± SD for *n* = 6. ** *p* < 0.01 versus the sham group; ^##^ *p* < 0.01 versus the OVX group.

The contractile response of bladder detrusor strips was assessed through electrical field stimulation (EFS), carbachol, and KCl treatment (Figure 2). In Figure 2A,D, the bladder strips from the OVX group exhibited higher contractile responses induced by EFS at 2, 8, and 32 Hz, as compared to the sham group (*p* < 0.001). Conversely, the OVX + E2 group showed lower contractile responses as compared to the OVX group (*p* < 0.001). Similar hyperactive responses were observed in the OVX group for carbachol (*p* < 0.001 vs. the sham group) (Figure 2B,D) and KCl (*p* < 0.001 vs. the sham group) (Figure 2C,D). E2 treatment improved bladder detrusor contractile responses, affecting synaptic transmission, receptor response, and smooth muscle contraction. These results indicated that the bladder of OVX rats exhibited hyperactive contractile responses, leading to bladder overactivity, which was ameliorated by E2 treatment.

### 2.5. Estradiol Treatment Regulated the Expression of ER, TGF-β and Smad Protein

**Figure 3:**
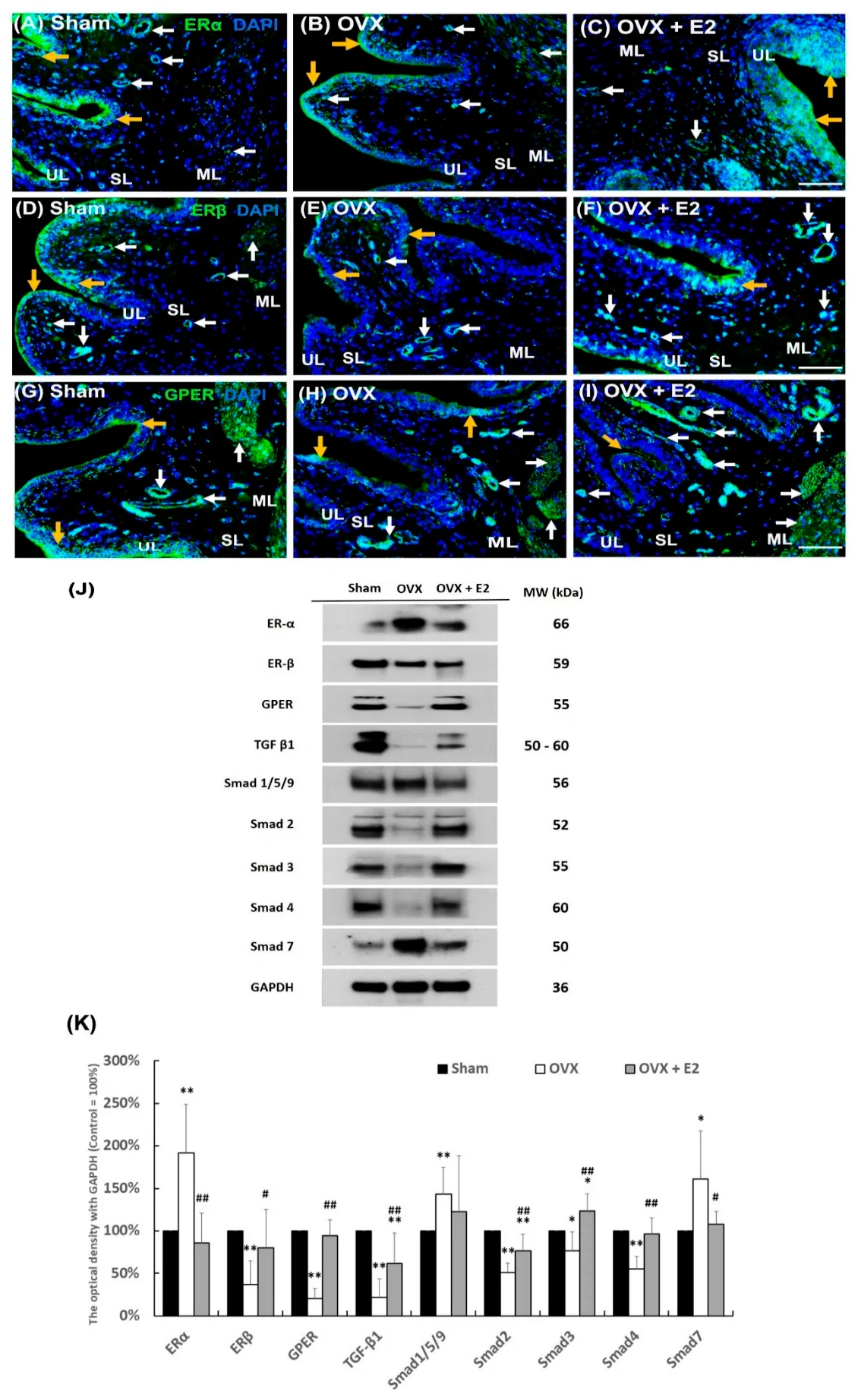
Estrogen receptor expression and TGF-β/Smad signaling in bladder tissues following ovariectomy and estradiol treatment. (**A**–**I**) Representative immunofluorescence staining of ERα (**A**–**C**), ERβ (**D**–**F**), and GPER (**G**–**I**) in bladder tissues from the Sham, OVX, and OVX + E2 groups. Yellow arrows representative immunoreactive signals in the urothelium, whereas white arrows indicated representative signals in the suburothelial and vascular regions. Nuclei were counterstained with DAPI. Scale bar = 100 μm. (**J**) Representative Western blot images of ERα, ERβ, GPER, TGF-β1, Smad1/5/9, Smad2, Smad3, Smad4, and Smad7, with GAPDH as the loading control. (**K**) Densitometric quantification of protein expression normalized to GAPDH. Data are presented as mean ± SD (*n* = 6 rats per group). Statistical comparisons were performed using one-way ANOVA followed by Bonferroni-adjusted post-hoc tests. * *p* < 0.05 and ** *p* < 0.01 versus the Sham group; ^#^ *p* < 0.05 and ^##^ *p* < 0.01 versus the OVX group. The original, uncropped Western blot images are available in the Supplementary Materials. Abbreviations: E2, estradiol; ER, estrogen receptor; GPER, G protein-coupled estrogen receptor; OVX, ovariectomy; TGF-β1, transforming growth factor-β1.

The expression levels of estrogen receptors (ERα, ERβ and GPER) and signaling pathway-related proteins (TGF-β1, Smad1/5/9, Smad2, Smad3, Smad4 and Smad7) were examined using immunostaining and Western blotting (Figure 3). In the sham group, immunostaining of ERα (Figure 3A), ERβ (Figure 3D), and GPER (Figure 3G) (yellow arrows) were broadly expressed in the urothelial layer (UL), vascular structures and interstitial cells of suburothelial layer (SL; stroma) and muscular layer (ML). In the OVX group, ERα expression (Figure 3B) was strong in the thinner UL, SL and ML, while the expressions of ERβ (Figure 3E), and GPER (Figure 3H) were reduced. In contrast, the expressions of ERα (Figure 3C), ERβ (Figure 3F), and GPER (Figure 3I) exhibited a thicker UL and more abundant vascular structures (neovascularization) and interstitial cells in the OVX + E2 group.

Quantitative Western blotting analysis (Figure 3J,K) revealed a significant upregulation of ERα in the OVX group compared to the sham group (*p* < 0.001), and a significant reduction in ERα expression in the OVX + E2 group compared to the OVX group (*p* < 0.001). Conversely, ERβ and GPER levels were markedly decreased in the OVX group compared to the Sham group (both *p* < 0.001), but were significantly increased in the OVX + E2 group compared with the OVX group (*p* = 0.038 and *p* < 0.001, respectively).

The expression levels of TGF-β1, Smad2, Smad3, and Smad4 were significantly reduced in the OVX group compared to the sham group (*p* < 0.001, *p* < 0.001, *p* = 0.016, and *p* < 0.001, respectively). These reductions were markedly reversed in the OVX + E2 group compared to the OVX group (*p* = 0.019, *p* = 0.006, *p* = 0.002, and *p* < 0.001, respectively). Smad7 expression was significantly increased in the OVX group (*p* = 0.024) and then significantly attenuated by E2 treatment in the OVX + E2 group (*p* = 0.049). Smad1/5/9 expression was significantly increased in the OVX group (*p* = 0.002), but no significant difference was observed between the OVX + E2 group and either the sham or the OVX groups. These findings suggested that E2 treatment improved bladder repair via the ER/TGF-β1/Smad signaling pathway in the pathogenesis of OHD-induced OAB-like storage phenotype.

### 2.6. Estradiol Supplementation Enhanced Autophagy Formation to Mitigate Bladder Damage Induced by OHD

**Figure 4:**
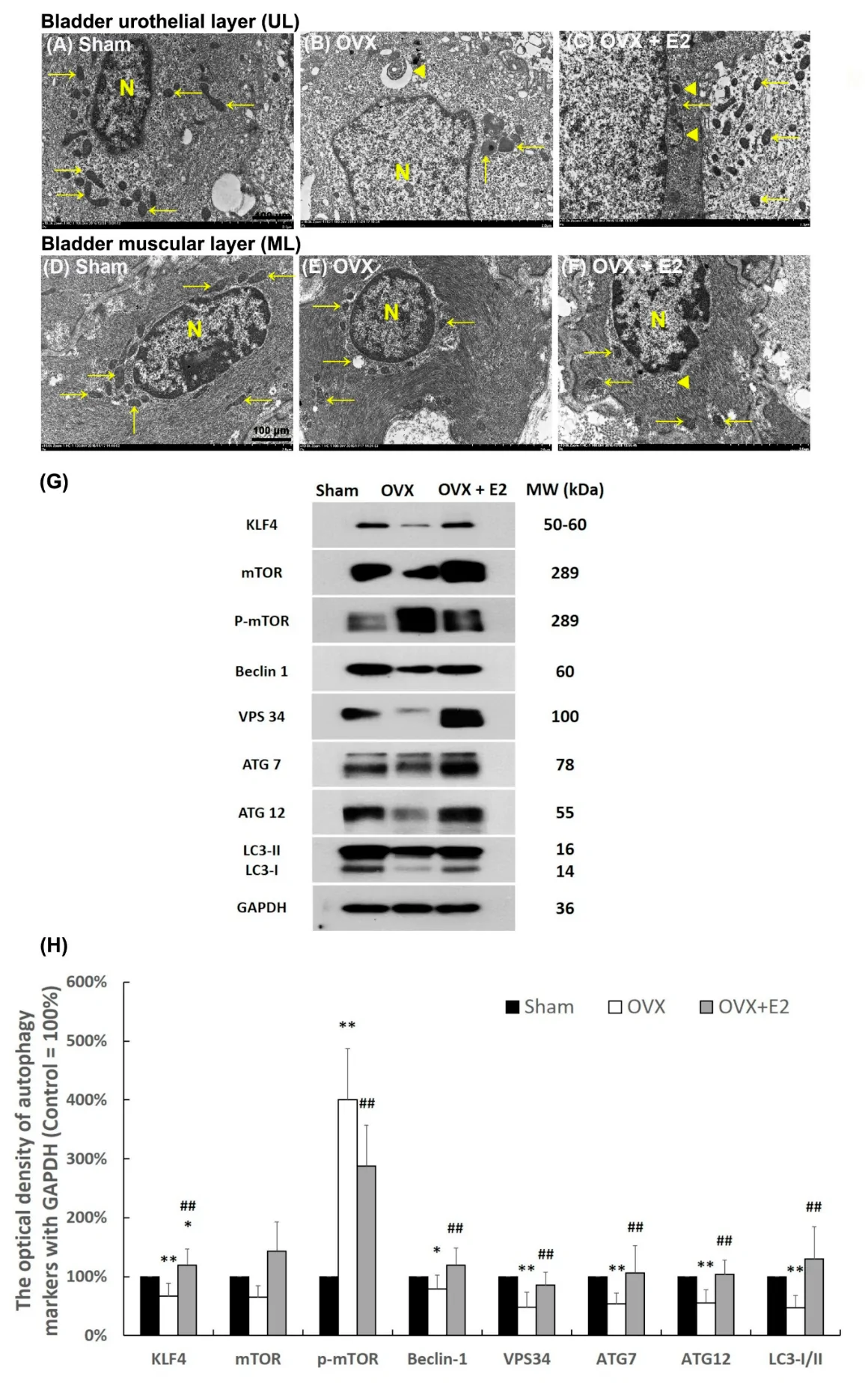
Estradiol restored ultrastructural integrity and autophagic protein expression in OVX bladders. Transmission electron microscopy (TEM) of mitochondrial ultrastructure and Western blot analysis of autophagy-related proteins. (**A**–**F**) Representative TEM micrographs of the bladder urothelial layer (UL; (**A**–**C**)) and muscular layer (ML; (**D**–**F**)) in Sham, OVX, and OVX + E2 groups. “N” indicates the cell nucleus. Yellow arrows highlight mitochondria; yellow arrowheads indicate double-membrane autophagosomes or autolysosomes engulfing degraded organelles. Scale bar = 100 nm. (**G**,**H**) Representative Western blot bands (**G**) and quantitative densitometric analysis (**H**) of KLF4, mTOR, p-mTOR, Beclin-1, VPS34, ATG7, ATG12, and LC3-II/I ratio normalized to GAPDH (expressed as % of Sham). Data are presented as mean ± SD (*n* = 6 per group). * *p* < 0.05 and ** *p* < 0.01 vs. Sham group; ^##^ *p* < 0.01 vs. OVX group. The original, uncropped Western blot images are available in the Supplementary Materials. Abbreviations: ATG, autophagy-related protein; GAPDH, glyceraldehyde 3-phosphate dehydrogenase; KLF4, Krüppel-like factor 4; LC3, microtubule-associated protein 1 light chain 3; ML, muscle layer; mTOR, mammalian target of rapamycin; N, nucleus; OVX, ovariectomy; p-mTOR, phospho-mammalian target of rapamycin; TEM, transmission electron microscopy; UL, urothelial layer; VPS34, vacuolar protein sorting 34.

TEM was used to visualize autophagic vacuoles and mitochondrial ultrastructure in bladder tissue (Figure 4A–F). In the sham group, the bladder urothelium exhibited intact nuclei (N) and well-preserved mitochondria (yellow arrows) with rare observation of autophagosome-like structures in UL (Figure 4A) and ML (Figure 4D). In contrast, the OVX group demonstrated significant ultrastructural damage, including swollen and degraded mitochondria (yellow arrows) and autolysosomes (yellow arrowhead) in UL (Figure 4B) and ML (Figure 4E), indicating mitochondrial dysfunction and impaired cellular homeostasis. The OVX + E2 group, however, displayed a restoration of cellular integrity, with numerous intact nuclei (N) and mitochondria (yellow arrows) present in UL (Figure 4C) and ML (Figure 4F). Notably, this group exhibited a significant increase in double-membrane autophagosomes in the cytosol (yellow arrowheads) that specifically engulfed degraded mitochondria, providing ultrastructural features consistent with mitophagy (Figure 4C,F).

Western blotting was used to analyze KLF4 and autophagy-related proteins, including mTOR, p-mTOR, ATG7, ATG12, Beclin-1, LC3-I, LC3-II, and VPS34 (Figure 4G,H). The expressions of KLF4 and autophagy-related proteins (Beclin-1, VPS34, ATG7, ATG12, and LC3-I/II) were significantly reduced in the OVX group compared to the Sham group (*p* < 0.001, *p* = 0.024, *p* < 0.001, *p* < 0.001, *p* < 0.001, and *p* < 0.001, respectively). Concurrently, the p-mTOR level was significantly elevated compared to the Sham group (*p* < 0.001). However, the expression levels of KLF4 (*p* < 0.001) and the aforementioned autophagy-related proteins (*p* = 0.008, *p* = 0.007, *p* = 0.010, *p* = 0.001, and *p* = 0.001, respectively) were significantly increased in the OVX + E2 group compared to the OVX group. There were no significant differences among the groups in the expression of total mTOR. These data indicated that estrogen deficiency suppressed autophagic protein expression by upregulating the expressions of p-mTOR, while E2 supplementation restored the expression of autophagic/mitophagic markers.

### 2.7. Estradiol Treatment Regulated the Expressions of miRNA-200 Family in Bladder

**Figure 5:**
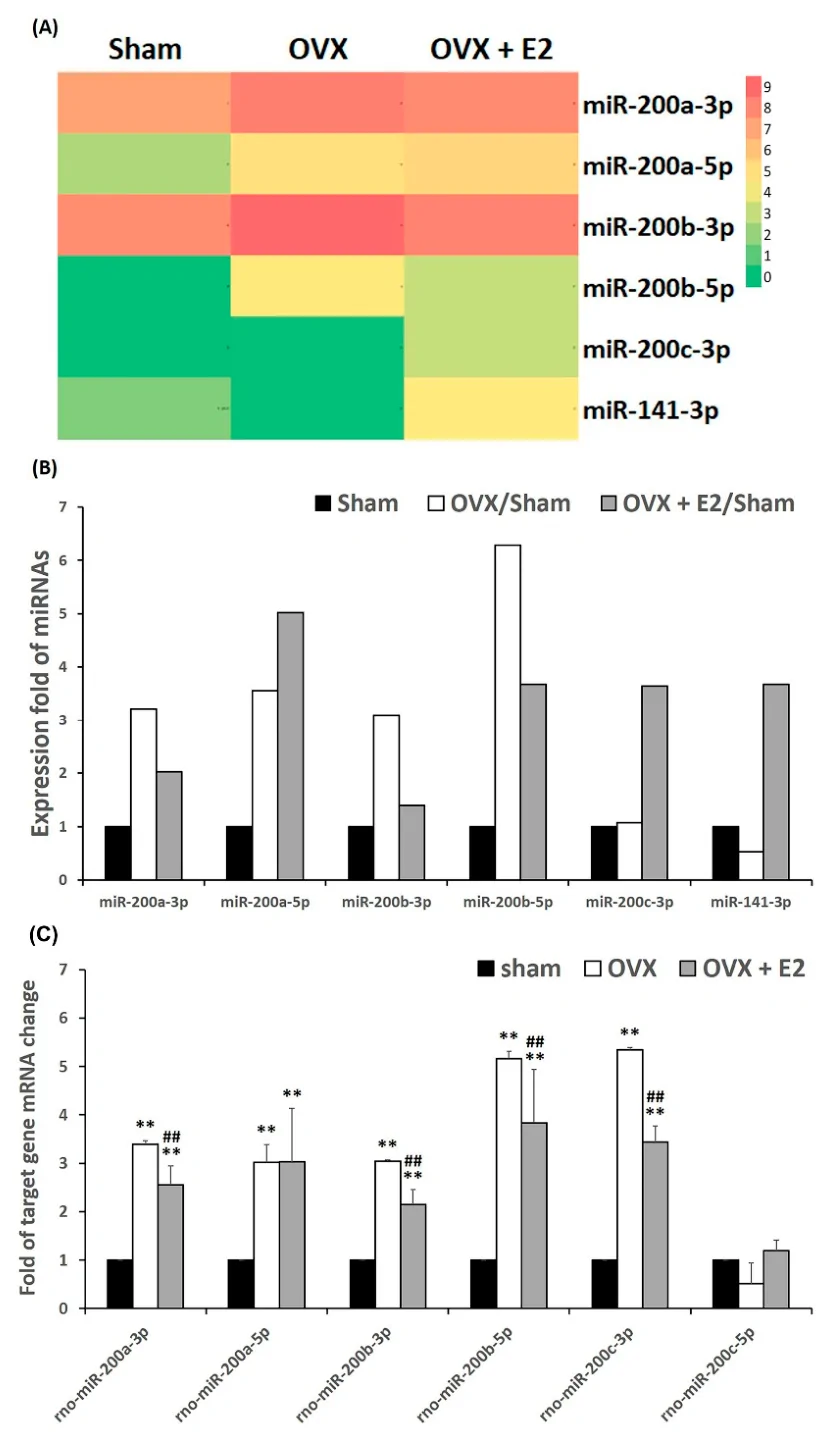
Estradiol treatment altered the expressions of miRNA-200 family in bladder. (**A**,**B**): The heatmap illustrated differentially expressed miRNAs identified across the sham group, the OVX group, and OVX + E2 group. (**B**) Relative expression analysis of miRNA-200 family was shown. In the OVX group, the expressions of miRNA-200a-3p, miRNA-200a-5p, miRNA-200b-3p and miRNA-200b-5p were upregulated compared to the sham group, while miRNA-141-3p was downregulated. In the OVX + E2 group, the levels of miRNA-200a-3p, miRNA-200b-3p and miRNA-200b-5p were reduced compared to the OVX group, whereas the levels of miRNA-200a-5p, miRNA-200c-3p and miRNA-141-3p were increased. (**C**): Relative expression analysis of miRNA-200 family by RT-qPCR. In the OVX group, the levels of miRNA-200a-3p, miRNA-200a-5p, miRNA-200b-3p, miRNA-200b-5p, and miRNA-200c-3p were significantly higher as compared to the sham group. Following E2 treatment, the levels of miRNA-200a-3p, miRNA-200b-3p, miRNA-200b-5p, and miRNA-200c-3p were reduced compared to the OVX group. Note: RT-qPCR, real-time quantitative polymerase chain reaction; OVX, ovariectomy; E2, estradiol. Data were represented as mean ± SD for *n* = 6. ** *p* < 0.01 versus the sham group; ^##^ *p* < 0.01 versus the OVX group.

Microarray and RT-qPCR analyses were conducted to investigate the expression of the miRNA-200 family in the bladder (Figure 5). Among the 262 distinct miRNAs analyzed, the miRNA-200 family exhibited pronounced changes between these groups. In the OVX group, miRNA-200a-3p, miRNA-200a-5p, miRNA-200b-3p, and miRNA-200b-5p were significantly elevated, while miRNA-141-3p was markedly decreased compared to the sham group (Figure 5A,B). E2 treatment in the OVX + E2 group notably reduced the levels of miRNA-200a-3p, miRNA-200b-3p, and miRNA-200b-5p compared to the OVX group. In contrast, miRNA-200a-5p, miRNA-200c-3p and miRNA-141-3p were markedly increased in the OVX + E2 group relative to the OVX group.

RT-qPCR analysis confirmed a significant upregulation of miRNA-200a-3p, miRNA-200a-5p, miRNA-200b-3p, miRNA-200b-5p, and miRNA-200c-3p in the OVX group compared to the sham group (all *p* < 0.001) (Figure 5C). Following E2 treatment, the levels of miRNA-200a-3p, miRNA-200b-3p, miRNA-200b-5p, and miRNA-200c-3p were markedly downregulated compared to the OVX group (*p* = 0.003, *p* < 0.001, *p* = 0.037, and *p* < 0.001, respectively). However, miRNA-200a-5p and miRNA-200c-5p exhibited no significant change after E2 supplementation. These findings highlight the sensitivity of the miRNA-200 family to E2 supplementation, particularly miRNA-200a and miRNA-200b, suggesting their involvement in estrogen-mediated physiological processes.

### 2.8. Proposed Potential Mechanism of Estradiol Enhanced Autophagy

**Figure 6:**
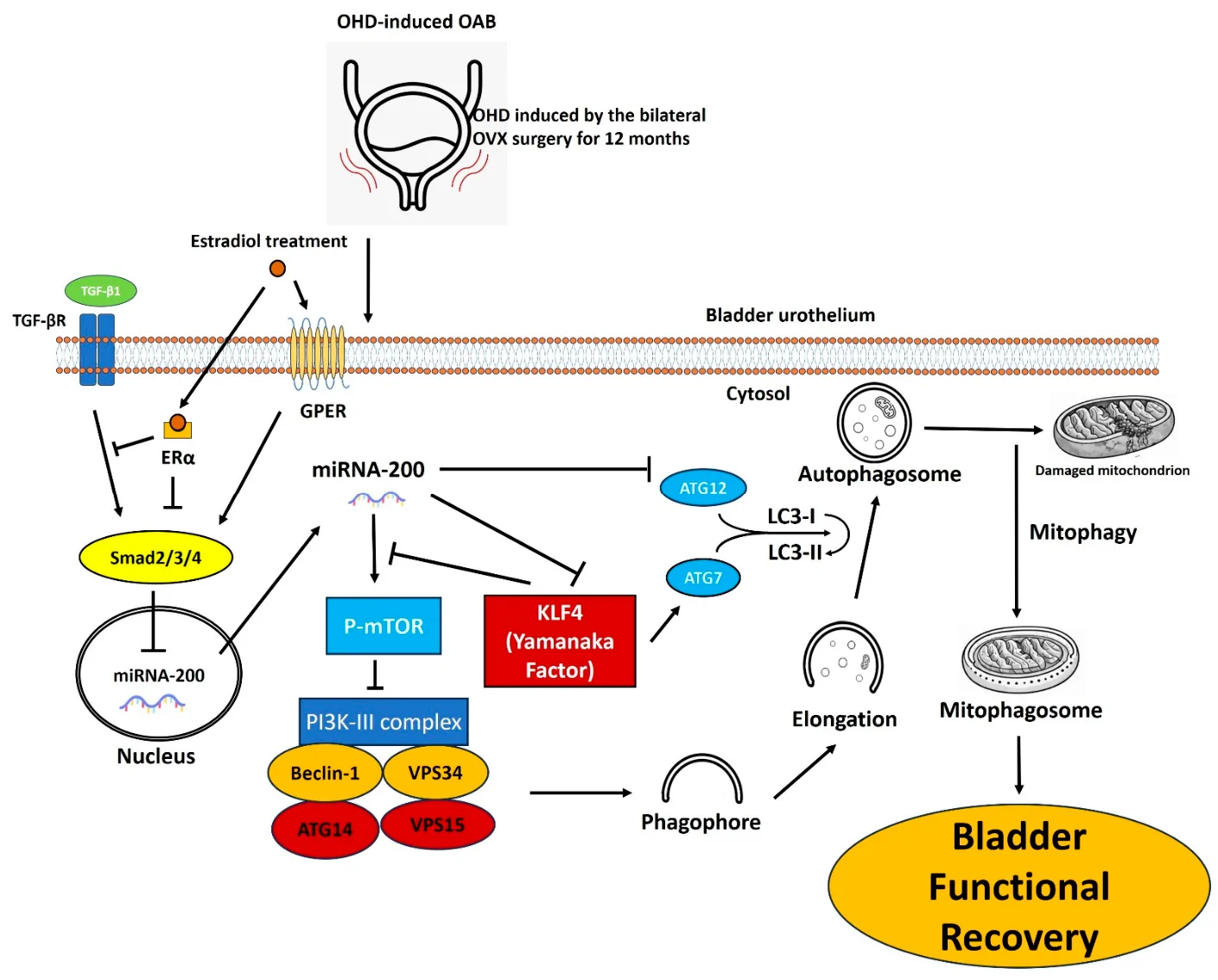
Schematic diagram of the proposed working model illustrated the potential relationships among estradiol, the TGF-β1/Smad/miRNA-200/KLF4/mTOR signaling pathway, mitochondrial quality control, and bladder function in the OVX-induced OHD rats. In this proposed model, OHD was associated with altered ER expression and reduced TGF-β1/Smad2/3/4-related signaling. These changes were accompanied by increasing expression of specific miRNA-200 family members (miRNA-200a-3p, miRNA-200b-3p, and miRNA-200b-5p), reducing KLF4 and autophagy-related protein expression, and elevating p-mTOR levels, collectively associated with impaired mitochondrial quality control and bladder overactivity. Conversely, E2 treatment partially reversed these alterations, with restoration of ERβ/GPER expression, TGF-β1/Smad-related signaling, reduced expression of specific miRNA-200 family members, increased KLF4 and autophagy-related protein expression, and reduced the p-mTOR levels. These coordinated changes were associated with improved mitochondrial ultrastructural integrity and bladder function. Solid lines indicated the associations supported by our experimental findings, whereas dashed lines represented the proposed or literature-supported mechanistic links that were not directly perturbed in the current in vivo model, including reported interactions between specific miRNA-200 family members and KLF4/ATG12 and the transcriptional regulation of ATG7 by KLF4. Arrows indicated stimulation, and T-bars indicated inhibition. Colors are used solely to distinguish different signaling components and molecular complexes for visual clarity. Note: ATG, autophagy-related gene; E2, estradiol; ERα, estrogen receptor α; ERβ, estrogen receptor β; GPER, G protein-coupled estrogen receptor 1; KLF4, Krüppel-like factor 4; LC3, microtubule-associated protein 1 light chain 3; mTOR, mammalian target of rapamycin; p-mTOR, phospho-mammalian target of rapamycin; OAB, overactive bladder; OHD, ovarian hormone deficiency; OVX, ovariectomy; PI3K, phosphoinositide 3-kinase; Smad, mothers against decapentaplegic homolog; TGF-β1, transforming growth factor-β1; TGFβR, TGF-β1 receptor; VPS34, vacuolar protein sorting 34.

Figure 6 illustrated the proposed mechanism by which E2 restored autophagic and mitophagic formation in OHD-induced OAB-like storage phenotype rats. In OVX rats, long-term OHD resulted in significant bladder dysfunction, characterized by increased micturition frequency, abnormal detrusor activity, and impaired contractility, whereas E2 treatment ameliorated OHD-induced bladder overactivity. In the OVX group, ERα expression was significantly increased, whereas ERβ and GPER expressions were significantly decreased compared with the sham group. E2 treatment markedly reversed these alterations, restoring ERβ and GPER expression. The expressions of signaling pathway-related proteins (TGF-β1, KLF4, Smad2, Smad3, and Smad4) and autophagy-related proteins (Beclin-1, VPS34, ATG7, ATG12, and LC3-I/II) were significantly reduced in the OVX group, accompanied by elevated p-mTOR levels, whereas E2 treatment restored these alterations. In parallel, the levels of miR-200a-3p, miR-200b-3p, miR-200b-5p, and miR-200c-3p were decreased following E2 treatment compared with the OVX group, accompanied by recovery of KLF4 expression and autophagy-related signaling. Conclusively, OHD-induced bladder dysfunction was associated with an imbalance in ER expression, characterized by upregulation of ERα and downregulation of ERβ and GPER, accompanied by suppression of TGF-β1/Smad signaling and upregulation of the miR-200 family. These alterations were associated with impaired autophagy-related signaling and bladder dysfunction, whereas E2 treatment reversed these changes. Taken together, these findings support a potential role for the ER/Smad/miR-200/KLF4/mTOR signaling pathway in the protective effects of E2 against OHD-induced bladder dysfunction.

## 3. Discussion

In the present study, we provided evidence that E2 ameliorated postmenopausal bladder overactivity, which was associated with improved mitochondrial quality con-trol features. We demonstrated that long-term OHD leads to significant bladder dys-function, characterized by increased micturition frequency, abnormal detrusor activity, and impaired contractility. At the molecular level, this dysfunction was associated with an imbalance in estrogen receptor expression (upregulated ERα, while ERβ and GPER were downregulated), which suppressed TGF-β1/Smad signaling and conse-quently led to the overexpression of the miRNA-200 family (miRNA-200a-3p, miR-NA-200b-3p, and miRNA-200b-5p). Crucially, we identified that the upregulation of the miRNA-200 family was associated with reduced KLF4 expression, elevated p-mTOR level, and ultrastructural features related with impaired mitochondrial qual-ity control in the bladder. Conversely, E2 supplementation reversed these pathological changes by modulating the ER/Smad/miRNA-200/KLF4/mTOR signaling pathway. E2 treatment was accompanied by improved mitochondrial ultrastructural integrity and increased expression of autophagy-related markers (p-mTOR, Beclin-1, VPS34, ATG7, ATG12, LC3-I/II), which might be correlated with the recovery of bladder func-tion. These findings highlighted the role of the ER-dependent regulation of mitophagy in maintaining bladder homeostasis.

The novelty of the present study lay in its integrated evaluation of bladder function, mitochondrial ultrastructure, miRNA expression, and autophagy-related signaling in a chronic OHD model. Previous studies have separately described estrogen-dependent changes in lower urinary tract structure and function and context-dependent interactions among estrogen receptors, Smad signaling, and autophagy. To our knowledge, however, the coordinated relationship among estrogen receptor subtype expression, TGF-β/Smad signaling, the miRNA-200/KLF4/mTOR signaling pathway, mitochondrial quality control, and bladder dysfunction has not been examined in an integrated manner in a long-term OVX model. In the present study, long-term OHD was associated with coordinated alterations in these functional, ultrastructural, and molecular parameters, which were attenuated or partially reversed following E2 treatment. These findings supported a proposed signaling framework linking estrogen status to mitochondrial quality control in the bladder. Nevertheless, because direct pathway manipulation and dynamic mitophagy-flux assays were not performed, this framework should be interpreted as a mechanistically informed and hypothesis-generating model rather than definitive evidence of a linear causal cascade.

The lower urinary tract, including the urethra and bladder, contains abundant ERs [12,32]. These receptors are widely distributed in the urothelium, interstitium, and smooth muscle layer, where they facilitate the maintenance of urogenital structure and function [33,34]. Previous studies indicate that ERβ expression remains relatively stable, whereas ERα is highly responsive to fluctuations in estrogen levels [35,36]. Consistent with these findings, we observed a marked upregulation of ERα following OVX, which was downregulated after E2 treatment. In the sham group, ERα, ERβ, and GPER were broadly expressed in the UL, microvasculature, and interstitial cells of the SL. However, the OVX group exhibited urothelial atrophy concomitant with strong ERα expression, while ERβ and GPER levels were significantly reduced. In contrast, E2 treatment promoted cell proliferation, restored urothelial thickness and promoted neovascularization in the SL, which correlated with the re-expression of ERβ and GPER. Notably, localized cell proliferation clusters were observed in the suburothelial layer of the OVX + E2 group, suggesting that E2-mediated signaling actively promotes tissue regeneration and structural repair.

OHD-related changes in bladder physiology, such as decreased urine flow and reduced bladder capacity, are frequently observed in elderly women and can contribute to urinary dysfunction [37]. Estrogen deprivation following menopause is strongly associated with the onset of OAB symptoms, emphasizing estrogen’s role in maintaining lower urinary tract health [38]. Hormone replacement therapy is used clinically to treat OAB and urinary incontinence in postmenopausal women [38,39]. Estrogen has been shown to improve bladder function by enhancing local blood flow and reducing urothelial atrophy [40,41]. At the molecular level, estrogen therapy has been shown to stimulate autophagic activity via ERα through a mechanism involving reactive oxygen species and the MEK/ERK signaling pathway [18]. In OVX rat models, selective ERβ activation improved bladder storage function, although ERα agonists had a more pronounced effect on voiding efficiency [42]. Clinically, topical estrogen therapy has been shown to increase ERβ expression in the bladder and alleviate OAB symptoms in postmenopausal women [43]. GPER is also involved in various physiological processes, including vasodilation and vascular remodeling, and may regulate cell proliferation [44]. In this study, the OVX group exhibited an upregulation of ERα and a downregulation of ERβ and GPER, suggesting that estrogen receptor signaling is critical for bladder function regulation.

Previous studies have shown that ERs can suppress TGF-β-induced activation of Smad3 [19]. In cyclophosphamide (CYP)-induced cystitis rats, TGF-β receptor inhibition improved bladder function [45]. In diabetic nephropathy, dysregulation of TGF-β/Smad3 signaling has been linked to autophagic markers downregulation [20]. Our previous research in a ketamine-induced cystitis rat model demonstrated that the autophagy inducer rapamycin increased the expression of autophagy-related proteins, supporting the therapeutic potential of enhancing autophagic markers in the bladder [16]. Smad7 is a known negative regulator of TGF-β1 signaling [46]. The ER/TGF-β1/Smad signaling pathway appears to play a central role in regulating autophagic activity under estrogen-deficient conditions. In this study, the OVX group exhibited decreased expression of TGF-β1, Smad2, Smad3, and Smad4, along with increased expression of Smad7 and p-mTOR. This was accompanied by a significant reduction in autophagy-related markers, including Beclin-1, VPS34, ATG12, ATG7, and LC3-I/II. E2 supplementation restored autophagic activity, as evidenced by the upregulation of these markers. Consistent with previous studies highlighting the role of estrogen in enhancing cell survival under stress [47,48], our findings suggested that the E2-mediated shift in receptor expression, specifically the recovery of ERβ and GPER, is essential for maintaining the TGF-β-dependent autophagic machinery, thereby ensuring cell survival and tissue homeostasis.

Beyond the signaling cascades, the preservation of mitochondrial integrity is fun-damental to bladder function. The accumulation of damaged mitochondria is a hall-mark of OHD-induced tissue injury, leading to excessive ROS production and bioener-getic failure [49]. In our study, TEM imaging provided direct visual evidence that E2 treatment not only reduced the number of swollen mitochondria but also facilitated their engulfment by autophagosomes. This suggested that the restoration of the ER/Smad/miRNA-200/KLF4 signaling pathway did not merely regulate protein expres-sion but was accompanied by ultrastructural features consistent with the clearance of damaged mitochondria. These findings were consistent with a potential role of im-proved mitochondrial quality control in the protective effects of E2.

MiRNAs function were critical post-transcriptional regulators of gene expres-sion [39]. Clinical studies have notably identified elevated levels of miRNA-200c-3p in patients with OAB [40], suggesting a potential pathogenic role. Mechanistically, the miRNA-200 family was closely linked to autophagic regulation through the suppres-sion of KLF4. Previous research demonstrated that miRNA-200b directly targeted KLF4 [41], which in turn activated mTOR to inhibit autophagy [42]. Furthermore, KLF4 acted as a transcriptional activator for ATG7 [50], while miRNA-200b can also directly target ATG12 [44]. In the present study, the OVX-induced upregulation of the miR-NA-200 family (miRNA-200a, -200b, and -200c) might be strongly correlated with the suppression of KLF4 and the inhibition of mitophagy. Conversely, E2 treatment suc-cessfully reversed these pathological changes by downregulating the miRNA-200 ex-pression, restoring KLF4 level, and promoting autophagy formation. In conclusion, our findings proposed a novel mechanism wherein E2 ameliorated the OHD-induced bladder overactivity by modulating the ER/Smad/miRNA-200/KLF4/mTOR sig-naling pathway. By restoring this pathway, E2 re-established mitochondrial function, highlighting its potential as a targeted therapeutic strategy for postmenopausal wom-en with OAB.

### Strengths and Limitations

A principal strength of this study was its integrated assessment of bladder function, mitochondrial ultrastructure, miRNA expression, and autophagy-related signaling in a chronic ovarian hormone deficiency model. To our knowledge, few studies have examined the coordinated relationship among estrogen receptor subtype expression, TGF-β/Smad signaling, the miR-200/KLF4/mTOR pathway, mitochondrial quality control, and bladder dysfunction within the same experimental framework. The combination of metabolic-cage assessment, cystometry, ex vivo contractility, TEM, miRNA profiling, RT-qPCR, Western blotting, and immunofluorescence provided complementary functional, ultrastructural, and molecular observations. In addition, the prolonged OVX protocol enabled evaluation of chronic ovarian hormone deficiency and age-associated bladder remodeling, which may provide greater relevance to long-standing postmenopausal bladder dysfunction than short-term OVX models. Nevertheless, this model does not fully reproduce the biological and clinical complexity of human menopause.

Despite these strengths, several limitations warrant consideration. First, while we identified the miRNA-200/KLF4 signaling pathway as a critical downstream effector of estrogen signaling, our findings were based on a rat model of OVX-induced OAB-like storage phenotype. Although this model closely mimics postmenopausal physiology, species-specific differences in receptor distribution and metabolic responses must be considered when translating these findings to humans. Second, our mechanistic insights into the ER/Smad/miR-200/KLF4/mTOR signaling pathway were primarily derived from observational correlations and in vivo pharmacological rescue. Although multi-level analyses (including urodynamics, TEM, miRNA sequencing, and Western blotting) demonstrated consistent, synchronized alterations across this pathway, we did not perform direct gain- or loss-of-function experiments such as using ER subtype-selective agonists/antagonists, synthetic miR-200 mimics/inhibitors, or cell-specific KLF4 depletion to unequivocally prove direct causality.

Previous bioinformatic and experimental studies supporteddations. Clinically, long-term systemic estrogen therapy KLF4 as a target of miR-200b in non-bladder cellular systems [30,31]. However, the present study did not perform a wild-type/mutant KLF4 3′-UTR reporter assay, AGO2-RNA immunoprecipitation, or miR-200/KLF4 perturbation experiments in bladder cells or tissues. Accordingly, the observed inverse expression pattern is consistent with, but does not independently establish, direct miR-200-mediated targeting of KLF4 in the bladder.

Fourth, dynamic flux turnover assays using lysosomal inhibitors were not performed in vivo, and highlighted this as a refined focus for future cell-based studies. Fifth, the assay-specific sample sizes were relatively small, ranging from four to six biological replicates per group, and no formal prospective power calculation based on a single prespecified primary endpoint was performed before initiation of the study. Although statistically significant between-group differences were detected for several key functional and molecular outcomes, the study may have had limited statistical power to detect modest biological effects, particularly for outcomes exhibiting greater interindividual variability. These findings should therefore be interpreted as exploratory and require confirmation in independently powered studies.

The E2 dose used in this study should be interpreted in the context of the achieved circulating hormone concentrations. Daily intramuscular administration of 30 μg/kg for 4 weeks restored serum E2 concentrations to the range observed in Sham rats at endpoint sampling, supporting its classification as a physiological replacement exposure within this rat model rather than a sustained supraphysiological regimen. However, because serial pharmacokinetic sampling was not performed, transient post-injection peaks cannot be excluded. Furthermore, differences in species, route of administration, metabolism, and pharmacokinetics preclude direct conversion of this regimen to a clinical HRT dose. Systemic E2 administration therefore served as a proof-of-concept intervention for testing the reversibility of OHD-associated bladder alterations and should not be interpreted as establishing the efficacy or safety of a corresponding regimen in postmenopausal women.

Finally, while systemic E2 administration successfully demonstrated the therapeutic potential of restoring the ER/Smad/miR-200/KLF4/mTOR signaling pathway in our rat model, it is crucial to distinguish these preclinical mechanistic findings from direct clinical recommendations. Clinically, long-term systemic estrogen therapy carried well-documented risks, including endometrial hyperplasia, breast neoplasia, and cardiovascular events. Therefore, systemic E2 in this study serves as a proof-of-concept tool to uncouple the cytoprotective signaling cascade rather than an unconditional endorsement for systemic HRT in all postmenopausal women with OAB. To bypass systemic estrogenic risks, future therapeutic translational research should focus on targeting non-hormonal downstream effectors within this pathway. These include the development of synthetic miR-200 antagomirs to derepress KLF4, small-molecule KLF4 upregulators, mTOR/mitophagy modulators (such as rapamycin derivatives), or local intravesical delivery formulations that directly target the bladder urothelium without systemic adverse effects.

## 4. Materials and Methods

### 4.1. Animal Induction and Preparation

**Figure 7:**
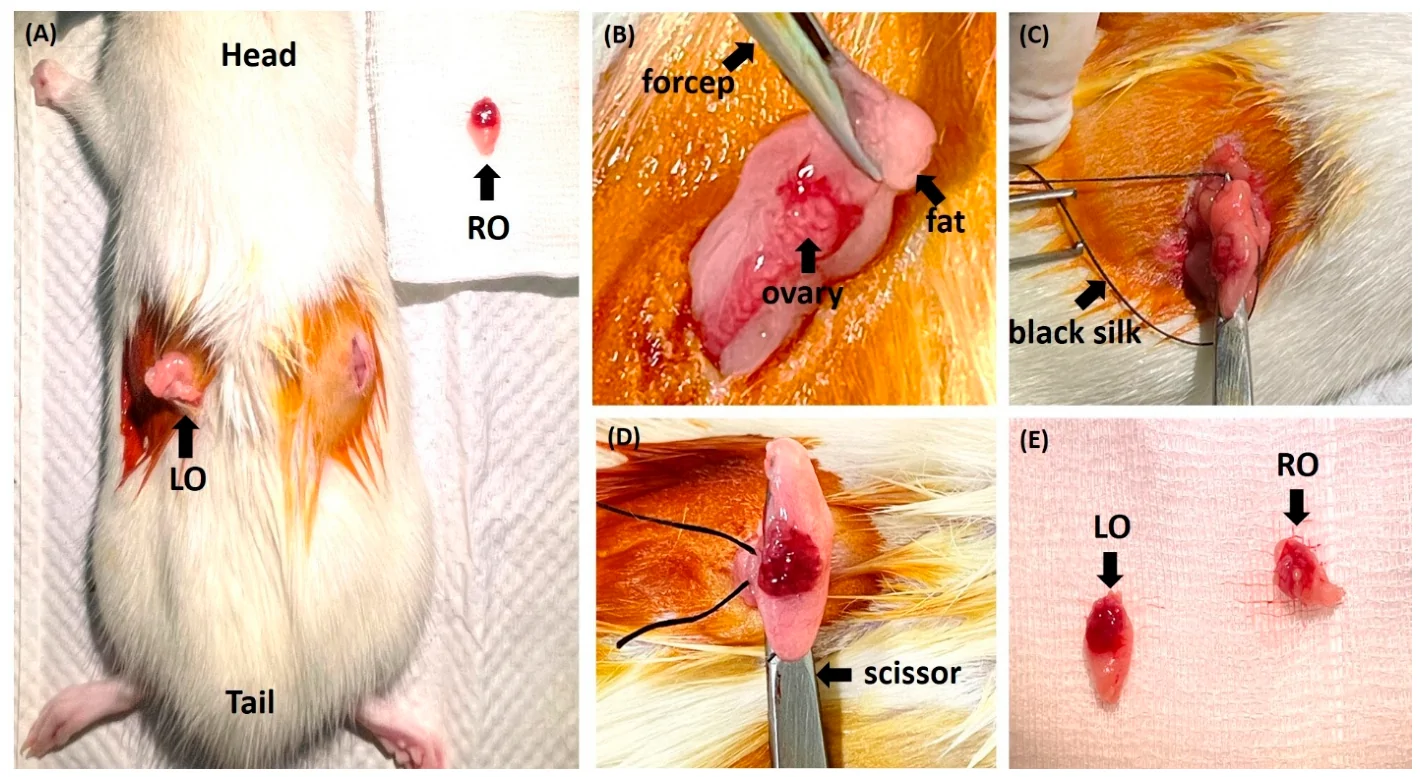
Experimental procedure for bilateral ovariectomy to induce ovarian hormone deficiency. (**A**) Bilateral ovaries were resected through dorsolateral incisions. (**B**) The ovarian fat pad was gently extracted using forceps to expose the ovary. (**C**) The oviduct and upper part of the uterine horn were retracted, and black silk knots were applied to delineate the tissue to be removed. (**D**) Ovaries were excised using scissors. (**E**) The resected ovaries. For sham operations, the same surgical steps were performed, but the ovaries were left intact. Note: OHD, ovarian hormone deficiency; LO, left ovary; RO, right ovary.

**Figure 8:**
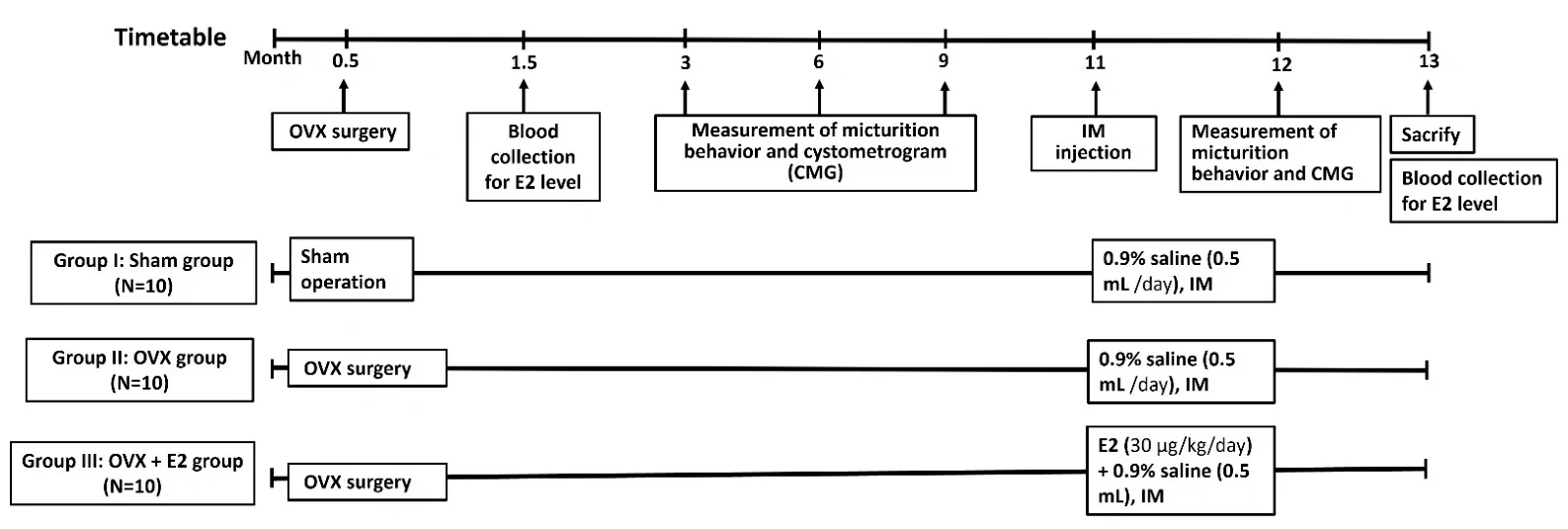
Experimental design.

This study was conducted and reported in accordance with the ARRIVE (Animal Research: Reporting of In Vivo Experiments) guidelines 2.0 for animal research. This experimental procedure received approval from the Committee for the Use of Experimental Animals at Kaohsiung Medical University (IACUC: KMUH-112227, KMUH-110187). Thirty female Sprague-Dawley rats (8–10 weeks old, weighing approximately 200–250 g at the start of the experiment) were obtained from the animal center of BioLASCO Taiwan Co., Ltd. (Taipei, Taiwan). They were divided into three groups: (A) the sham group: the rats received sham operation, (B) the OVX group: OHD induced by bilateral OVX for 11 months, and (C) the OVX + E2 group: the rats with OHD maintained for 11 months followed by daily intramuscular injection of E2 (E2, 30 μg/Kg) (Ta Fong pharmaceutical Co., Ltd., Changhua, Taiwan). The OVX surgery was performed under halothane anesthesia. Bilateral ovaries were excised through 1 cm incisions made on parallel bilateral sides of the spine to induce OHD (Figure 7). The 11-month post-OVX period was chosen to establish chronic, age-related postmenopausal bladder dysfunction, as previously validated [51,52]. The E2 regimen of 30 μg/kg/day for 4 weeks was selected on the basis of previous OVX rodent studies [53,54,55] and was intended to provide systemic estrogen replacement in this animal model. The adequacy of hormone replacement was evaluated by measuring serum E2 concentrations before and after the treatment period [53,54,55]. Specifically, animals underwent bilateral OVX for 12 months prior to receiving 1 month of daily E2 treatment or vehicle, completing a total post-OVX duration of 12 months. Estradiol (E2, 30 μg/kg/day) was diluted in 0.9% normal saline (0.5 mL) and administered via daily intramuscular (IM) injection for 4 weeks. The sham rats were treated with sham operation and IM injection of volume-matched 0.9% saline (0.5 mL). In addition, the OVX rats received volume-matched 0.9% saline (0.5 mL)-only IM injections on the same schedule and for the same duration as the OVX + E2 rats (Figure 8). To ensure equivalent handling and eliminate vehicle- or stress-induced confounders, rats in the Sham group and the OVX group received intramuscular (IM) injections of 0.9% saline (0.5 mL) alone on the exact same schedule and duration. Serum E2 concentration was checked. At the conclusion of the 1-month E2 treatment after 11 months post-OVX, animals were individually placed in metabolic cages for 24 h to evaluate micturition patterns and physical indicators. Water consumption, total urine output, and voiding frequency were systematically recorded during this 24 h period prior to in vivo cystometric testing and subsequent sacrifice for tissue harvest.

### 4.2. Experimental Design, Attrition, Tissue Allocation, and Assay-Specific Sample Sizes

Thirty female Sprague-Dawley rats were initially allocated to the Sham, OVX, and OVX + E2 groups, with 10 animals assigned to each group. The initial group size was selected to account for potential attrition during the prolonged experimental protocol, which comprised an 11-month post-OVX period followed by 4 weeks of E2 or vehicle treatment.

During the 12-month experimental period, 12 animals died during long-term follow-up or in association with anesthesia-related adverse events. The losses comprised four animals from each experimental group, leaving six surviving animals per group for the final evaluations.

The final biological sample sizes were *n* = 6 per group for the 24 h micturition assessment, cystometry, Western blotting, RT-qPCR, immunostaining, and transmission electron microscopy. For the ex vivo detrusor strip contractility assays, technically valid recordings were obtained from four animals per group. The reduced assay-specific sample size for contractility resulted from technical recording limitations, such as failure of some bladder strips to achieve or maintain stable baseline tension, and did not represent additional animal mortality or exclusion from other analyses.

### 4.3. Estradiol Level Measurement

After OVX surgery for four weeks, serum E2 concentration was checked. Serum E2 levels were measured according to previously described methods [51,52,56,57]. Blood samples were collected from the tail vein under anesthesia and subsequently centrifuged at 4 °C. The blood samples were processed according to the manufacturer’s protocol for the E2 ELISA kit (catalog no. # 582701, Cayman Chemical Co., Ann Arbor, MI, USA) according to the manufacturer’s instructions. Briefly, serum samples were added to antibody-coated microtiter wells along with Estradiol AChE Tracer and Estradiol Antiserum. After incubation and washing, Ellman’s Reagent was added to each well and developed in the dark. The concentration of E2 was measured using an ELISA reader (Bio-Tek ELX 800, BioTek, Bad Friedrichshall, Germany). Average absorbance values were computed for both standard and experimental serum samples across different groups.

### 4.4. Measurements of Micturition Volume and Frequency

To minimize stress-induced artifactual voiding, rats were housed individually in the metabolic cages for a 24 h adaptation period with free access to food and water prior to formal recording. The micturition behavior and physical indicators were evaluated using KDS-TL380 metabolic cages (Lab Products, Rockville, MD, USA) in accordance with methods previously described [5,6,51]. Voided urine was directed through a funnel into a collection container connected to an MLT0380 force transducer (ADInstruments, Colorado Springs, CO, USA). Continuous signals were acquired over the subsequent 24 h observation period using a PowerLab data acquisition system paired with LabChart software (version 7, ADInstruments, Windows 7 system). The primary parameters recorded and analyzed included 24 h water consumption, total urine output, voiding frequency (number of voids/24 h), and mean single voided volume.

### 4.5. Cystometrograms (CMGs)

The CMG measurement was conducted following the protocol outlined in a prior study [5,6,51]. Cystometry was performed under Zoletil anesthesia (Virbac, Taipei, Taiwan) 1 mg/kg, intraperitoneal injection). Zoletil was selected in accordance with our previously established and institutionally approved cystometry protocol. The adequacy of anesthesia was assessed using actual monitoring criteria, such as absence of the pedal withdrawal reflex and regular respiratory pattern. After catheter placement, recordings were initiated following a stabilization period. The same anesthetic dose, route, monitoring procedure, stabilization period, infusion rate, and recording conditions were used for all animals. After administering analgesia, a PE50 tube was inserted via the urethra into the bladder to empty its contents. 0.9% normal saline was infused into the bladder via the urethral catheter at a steady rate of 0.08 mL/min. The tube was connected to a pressure transducer in order to measure intravesical pressure. To ensure stable physiological conditions, at least five consecutive cycles of bladder filling and voiding were recorded initially. Physiological signals were amplified using ML866 (PowerLab, ADInstruments) and recorded with LabChart 7 (ADInstruments, Windows 7 system).

After stabilization, at least five consecutive filling–voiding cycles were recorded and averaged for each animal. Operational definitions for key functional parameters were specified as follows: Peak Micturition Pressure ($P_{\max}$): defined as the maximum intravesical pressure recorded during an active voiding contraction. Non-Voiding Contractions (NVCs): spontaneous intravesical pressure rises exceeding $2{\;\mathrm{cmH}}_{2}O$ above baseline during the filling phase without urine evacuation. Functionally, an OAB-like storage phenotype was characterized by detrusor overactivity, manifested by increased micturition frequency, elevated NVC frequency, and reduced bladder capacity. Because bladder compliance (ΔV/ΔP\Delta V/\Delta PΔV/ΔP) was not calculated, it was not included as a measured endpoint.

### 4.6. Bladder Muscle Strips to Assess Bladder Contractility

Following completion of cystometry, the animals were euthanized and the bladders were immediately harvested. Bladder tissues from four animals per group were used for ex vivo contractility analysis. The bladder contractility was measured in accordance with the method previously described [5,6,51]. Bladder longitudinal strips measuring approximately 5 × 15 mm^2^ were obtained from the bladder, spanning from the trigone to the dome. The strips were placed in oxygenated Krebs–Henseleit solution at 37 °C for 30 min. An initial resting tension of 2 g was applied for 30 min. Between challenges, baths were washed 3–4 times with fresh oxygenated Krebs solution and allowed to return toward baseline for 15 min. Neurogenic contractile responses were elicited using EFS parameters (2, 8 and 32 Hz). Muscarinic receptor-mediated responses were generated via cholinergic agonist carbachol (20 μM) (Catalog no. CAS 51-83-2 Sigma-Aldrich, Darmstadt, Germany) addition. Receptor-independent membrane depolarization was induced by adding 120 mM KCl (Catalog no. 529552, Sigma-Aldrich, Darmstadt, Germany). Between each functional challenge (EFS, carbachol, and KCl), the organ baths were thoroughly washed out 3–4 times with fresh Krebs solution, followed by a 15 min resting interval to allow baseline tension to completely recover. All contractile forces were continuously recorded using isometric force transducers connected to a PowerLab acquisition system (ADInstruments) and normalized to the peak tension elicited by 120 mM KCl. The collected data were digitized and analyzed using the Grass POLYVIEW A-D conversion system (Grass Instrument Co., Warwick, RI, USA).

### 4.7. Immunofluorescence Staining to Spot Protein Expression

Bladder tissues were fixed overnight in 4% paraformaldehyde prepared in 0.1 M phosphate-buffered saline (PBS, pH 7.4), followed by paraffin embedding and sectioning into 5 μm-thick slices. Immunofluorescence staining was performed to determine the precise localization of the target protein, in accordance with protocols described in previous studies [5,6,51]. Bladder tissue sections were blocked with 10% normal goat serum (NGS) in PBS containing 0.5% Triton X-100 (Merck, Darmstadt, Germany) for 1 h at room temperature. Following blocking, the sections were incubated overnight at 4 °C with primary antibodies against estrogen receptors: ERα (Abcam, Cambridge, UK, rabbit monoclonal, 1:100, catalog no. ab32063), ERβ (Affinity Biosciences, Cincinnati, OH, USA, rabbit polyclonal, 1:25, catalog no. AF6469), and GPER (Abcam, Cambridge, UK, rabbit monoclonal, 1:100, catalog no. ab26033). The sections were subsequently rinsed with PBS containing 0.5% Triton X-100 for 15 min, then incubated with secondary antibodies (1:800 dilution; Invitrogen, Cat# G-21234, Carlsbad, CA, USA) at room temperature for 1 h. Following PBS washes, nuclei were counterstained with DAPI, and the sections were mounted using ProLong™ Gold Antifade Reagent (Invitrogen, Cat# P36934, Carlsbad, CA, USA). A negative control was included by omitting the primary antibody to assess nonspecific immunostaining.

### 4.8. Western Blot Analysis to Assess Protein Expression Levels

Frozen bladder tissues were homogenized in lysis buffer containing 50 mM Tris (pH 7.5) and 5% Triton X100, supplemented with Halt Protease Inhibitor Cocktail (Pierce, Rockford, IL, USA) on ice. The homogenates were then centrifuged at 14,000× *g* at 4 °C for 20 min. Equal amounts of total protein (20 μg) were separated on 12% SDS polyacrylamide gels and subsequently transferred to PVDF membranes. The membranes were blocked with 5% non-fat milk and then incubated with primary antibodies targeting various markers, including estrogen receptors (ERα, ERβ and GPER), TGF-β, Smad (Smad1/5/9, Smad2, Smad3, Smad4, Smad7), transcription factor (KLF4) and autophagic-related proteins (mTOR, p-mTOR, beclin-1, VPS34, ATG7, ATG12, LC3-I and LC3-II). Glyceraldehyde-3-phosphate dehydrogenase (GAPDH) was used as a reference for normalization. Visualization of the blots was performed using enhanced chemiluminescence (ECL), and images were captured on Biomax L film (Carestream Health, Rochester, NY, USA). Negative controls, omitting the primary antibody, were included in each experiment. Western blot analyses were replicated three times, and the resulting bands were quantified using Image J software (version 1.54g). Further details of the Western blotting procedure can be found in Appendix B.

### 4.9. Transmission Electron Microscopy (TEM)

TEM was employed to examine the ultrastructure of bladder tissues in accordance with the method previously described [16,51]. The tissues were collected and immersed overnight in 0.1 M phosphate-buffered saline containing 2% paraformaldehyde and 2.5% glutaraldehyde. Following this, the samples were fixed with 2% osmium tetroxide at 4 °C for 90 min, embedded in epon, and sectioned into 70 nm slices using an automatic ultramicrotome (Reichert Ultracut E, Vienna, Austria). The ultrastructural morphology was then analyzed using a transmission electron microscope (JEM2000 EXII; Jeol Ltd., Tokyo, Japan).

### 4.10. MiRNA-200 Family Extraction from Bladder Tissue

The expression levels of miRNA-200 family, including miRNA-200a, miRNA-200b, miRNA-200c, miRNA-141, and miRNA-429, were measured in accordance with the method previously described [58,59]. MiRNA-200 family was isolated from rat bladder tissue using FastPure Cell/Tissue Total RNA Isolation Kit (Catalog No. RC101, Vazyme Biotech, Nanjing, China) according to the manufacturer’s protocol. Subsequently, the concentration of microRNA was determined using the Qubit microRNA Assay Kit (Catalog no.: Q32880, Thermo Fisher Scientific, Waltham, MA, USA) on a Qubit Fluorometer, and the length distribution of microRNA (~22 nucleotides) was assessed by microfluidic electrophoresis using the RNA Kit (Catalog no.: 292-27913-91) on a MultiNA MCE-202 (Shimadzu, Kyoto, Japan).

### 4.11. Construction of the miRNA 200 Family Library for Next-Generation Sequencing

Each sample used 10 ng of miRNA 200 family, quantified using the Qubit microRNA Assay Kit, to construct the miRNA library with the VAHTS Small RNA Library Prep Kit for Illumina and VAHTS Small RNA Index Primer Kit for Illumina (Catalog no.: NR801-01, Vazyme Biotech, Nanjing, China). The correctly barcoded library amplicons were approximately 142 bp in length. Target fragments were excised from a 4% agarose gel and purified using the FastPure Gel DNA Extraction Mini Kit (Catalog no.: DC301-01, Vazyme Biotech). Microfluidic electrophoresis was used to quantify the concentration and assess the length distribution of the eluted barcoding adapter-ligated library (142 bp) using the DNA-2500 Kit on the MultiNA MCE-202 instrument (Shimadzu, Japan). The pooled library, qualified at 4nM concentration, underwent sequencing on an Illumina NextSeq 500 (single-end, 75 cycles) instrument (Illumina, San Diego, CA, USA).

### 4.12. Quantification of Bladder miRNAs (RT-qPCR)

Total RNA (20 ng) was reverse-transcribed using the Quick-RNA Miniprep Plus kit (Category No.: R1057, Zymo Research, Irvine, CA, USA) and miRNA-specific stem-loop primers, following the manufacturer’s instructions (Thermo Fisher Scientific, Waltham, MA, USA). The relative levels of individual miRNAs were amplified using TaqMan MicroRNA assays on a 7900HT Sequence Detection System (Thermo Fisher Scientific, Waltham, MA, USA). The amplification program included an initial denaturation at 95 °C for 10 min, followed by 40 cycles of 95 °C for 15 s and 60 °C for 1 min. Specific TaqMan probes were used for mature miRNA sequences: rno-miRNA-200a-3p (000502), rno-miRNA-200a-5p (464052_mat), rno-miRNA-200b-3p (001800), rno-miRNA-200b-5p (002274), rno-miRNA-200c-3p (463287_mat), rno-miRNA-200c-5p (462130_mat), and U6 snRNA (001973) as an internal control (Appendix A). The relative expression level of each miRNA-200 member was calculated using the comparative Ct method, 2^(−ΔCt)^, where ΔCt = Ct of the miRNA-200 member—Ct of U6 snRNA.

### 4.13. Statistical Analysis

Data are presented as the mean ± standard deviation (SD). The individual rat was designated as the biological experimental unit. Technical replicates obtained from the same animal were averaged before group-level analysis and were not treated as independent biological observations. Repeated measurements across EFS frequencies were retained as within-subject observations and analyzed using a repeated-measures model. The final assay-specific biological sample sizes were *n* = 6 per group for 24 h micturition assessment, cystometry, Western blotting, and RT-qPCR, and *n* = 4 per group for the ex vivo detrusor strip contractility assays. Immunostaining and transmission electron microscopy (TEM) were used for representative qualitative assessments and were not included in inferential statistical testing.

Outcomes consisting of a single measurement per animal across the three experimental groups, including metabolic-cage parameters, cystometric indices, Western blot densitometry, and RT-qPCR expression levels, were analyzed using one-way analysis of variance (ANOVA). When the omnibus ANOVA was statistically significant, the two prespecified pairwise comparisons—Sham versus OVX and OVX versus OVX + E2—were performed using Bonferroni-adjusted post hoc tests. The Bonferroni adjustment was applied to the two pairwise comparisons within each outcome.

Contractile responses measured repeatedly across EFS frequencies of 2, 8, and 32 Hz were analyzed using two-way repeated-measures ANOVA, with experimental group as the between-subject factor and stimulation frequency as the within-subject factor. The main effects of experimental group and stimulation frequency and the group-by-frequency interaction were evaluated. Sphericity was assessed using Mauchly’s test, and the Greenhouse–Geisser correction was applied when the sphericity assumption was violated. When appropriate, subsequent pairwise comparisons were adjusted using the Bonferroni procedure. Contractile responses to single-concentration challenges with 20 μM carbachol and 120 mM KCl were analyzed using one-way ANOVA followed by Bonferroni-adjusted pairwise comparisons.

Absolute bladder weight was analyzed using analysis of covariance (ANCOVA), with experimental group as the fixed factor and body weight as the covariate. The group-by-body-weight interaction was evaluated before the main ANCOVA analysis to assess the homogeneity-of-regression-slopes assumption. Exact two-sided *p*-values are reported wherever available from the statistical output, whereas values below 0.001 are reported as *p* < 0.001. An omnibus two-sided *p*-value < 0.05 was considered statistically significant. For post hoc comparisons, a Bonferroni-adjusted two-sided *p*-value < 0.05 was considered statistically significant. Statistical analyses were performed using SPSS version 26.0 (IBM Corp., Armonk, NY, USA).

## 5. Conclusions

In summary, this study elucidates the evidence for a potential molecular mechanism associated with the protective effects of E2 against OHD-induced bladder overactivity. We demonstrated that E2 treatment may restores the critical balance of estrogen receptors—specifically by rescuing ERβ and GPER expression—which activates the TGF-β/Smad signaling pathway and reduced expression of the miRNA-200 family. This suppression relieves the inhibition of KLF4, thereby preventing mTOR hyperactivation and re-establishing mitochondrial quality control (mitophagy). Consequently, these molecular changes may contribute to the clearance of damaged mitochondria preserves bladder structural integrity and function. These findings highlight the pivotal role of the ER/Smad/miRNA-200/KLF4 signaling pathway in maintaining urothelial homeostasis and suggest that targeting this autophagic pathway represents a promising therapeutic strategy for the management of postmenopausal overactive bladder.

## Figures and Tables

**Table 1:** Serum parameters, physical indicators and urodynamic parameters for the different groups.

	Sham	OVX	OVX + E2
No. rats	6	6	6
Serum estradiol conc. (pg/mL) before treatment	27.05 ± 6.07	7.53 ± 2.35 **	7.08 ± 2.56 **
Serum estradiol conc. (pg/mL) after treatment	30.94 ± 3.34	7.20 ± 1.79 **	28.49 ± 4.28 ^##^
Physical indicator			
Water intake (mL/24 h)	30.63 ± 6.97	35.50 ± 6.82	37.50 ± 8.68
Urine output (mL/24 h)	19.52 ± 4.03	18.13 ± 2.42	21.03 ± 3.41
Body weight (g)	394.75 ± 35.93	520.88 ± 52.51 **	446.25 ± 44.59 *^,^^##^
Bladder weight (mg)	170.00 ± 35.05	160.00 ± 10.69	155.00 ± 14.14
The ratio of bladder weight (mg)/body weight (g)	0.43 ± 0.10	0.31 ± 0.02 **	0.35 ± 0.04 *^,##^
Urodynamic parameters (Frequency of micturition)			
Frequency (No. voids/24 h)	9.63 ± 1.92	19.50 ± 1.93 **	12.63 ± 2.50 *^,##^
Voided volume (mL)	2.07 ± 0.43	0.93 ± 0.09 **	1.69 ± 0.24 *^,##^
Urodynamic parameters (CMG)			
Contraction frequency (No. voids/1 h)	3.25 ± 0.46	7.88 ± 1.55 **	3.88 ± 0.35 **^,##^
Peak micturition pressure (mmH_2_O)	29.36 ± 4.63	37.52 ± 6.54 **	26.93 ± 5.30 ^##^
No. of non-voiding contractions between micturition (No. voids/h)	0.00 ± 0.00	6.25 ± 1.39 **	1.75 ± 0.46 **^,##^
Calculated voided volume (mL)	1.50 ± 0.19	0.64 ± 0.16 **	1.25 ± 0.14 **^,##^

Note: E2, Estradiol; OVX, bilateral ovariectomy; OHD, ovarian hormone deficiency; OVX + E2, OHD status for 11 months, followed by daily E2 injection for one month. Values are the means ± SD. * *p* < 0.05 and ** *p* < 0.01 versus the sham group; ^##^ *p* < 0.01 versus the OVX group.

## Data Availability

The original contributions presented in this study are included in the article/Supplementary Materials. The original, uncropped Western blot images, including corresponding molecular weight markers and GAPDH loading controls for estrogen receptors (ERα, ERβ, GPER), TGF-β1/Smad signaling proteins, KLF4, and autophagy-related markers (mTOR, p-mTOR, Beclin-1, VPS34, ATG7, ATG12, LC3-I/II), are available as Supplementary Materials.

## Supplementary Materials

The following supporting information can be downloaded at https://www.mdpi.com/article/10.3390/ijms27177529/s1.

## Appendix A. Mature miRNA Sequences, miRBase Accession Numbers, and TaqMan Assay IDs Used for RT-qPCR Analysis

**Table A1:** List of candidate miRNAs, NCBI/miRBase accession numbers, and corresponding TaqMan assay IDs used for miRNA expression analysis.

Candidate Name	NCBI/miRbase Accession Number	TaqMan Assay ID
U6 snRNA	NR_004394	001973
Rno-miR200a-3p	MIMAT0000874	000502
Rno-miR200a-5p	MIMAT0017151	464052_mat
Rno-miR200b-3p	MIMAT0000875	001800
Rno-miR200b-5p	MIMAT0017152	002274
Rno-miR200c-3p	MIMAT0000873	463287_mat
Rno-miR200c-5p	MIMAT0017150	462130_mat

## Appendix B. Other Materials and Procedures Used in Western Blot Experiments

Membranes were incubated with the indicated primary antibodies described as follows: Estrogen receptor α (ERα; Abcam, Cambridge, UK, rabbit monoclonal, 1:1000, MW: 66 kDa, catalog No. ab32063), ERβ (ERβ (Affinity Biosciences, Cincinnati, OH, USA, rabbit polyclonal, 1:1000, MW: 59 kDa, catalog no. AF6469), G protein-coupled estrogen receptor 1 (GPER; Abcam, Cambridge, UK, rabbit monoclonal, 1:1000, MW: 55, catalog no. ab26033), transforming growth factor-β1 (TGF-β1; R&D systems, Minneapolis, MN, USA, mouse monoclonal, 1:1000, MW: 25 kDa, catalog no. MAB240), mothers against decapentaplegic homolog 1/5/9 (Smad1/5/9; Affinity Biosciences, Cincinnati, OH, USA, rabbit polyclonal, 1:1000, MW: 56 kDa, catalog no. AF0614), Smad2 (Thermo Fisher Scientific, Waltham, MA, USA, rabbit polyclonal, 1:1000, MW: 52 kDa, catalog no. 51-1300), Smad3 (Thermo Fisher Scientific, Waltham, MA, USA, rabbit polyclonal, 1:1000, MW: 55 kDa, catalog no. PA5-34774), Smad4 (Thermo Fisher Scientific, Waltham, MA, USA, rabbit polyclonal, 1:1000, MW: 60 kDa, catalog no. MA5-50750), Smad7 (R&D systems, Minneapolis, MN, USA, mouse monoclonal, 1:1000, MW: 50 kDa, catalog no. MAB2029), Krüppel-like factor 4 (KLF4; Proteintech, Rosemont, IL, USA, rabbit polyclonal, 1:1000, MW: 50–60 kDa, catalog No. 11880-1-AP), mTOR (Cell Signaling Technology, Danvers, MA, USA, rabbit monoclonal IgG, 1:1000, MW: 289 kDa, catalog no. 2983), Phospho-mTOR (p-mTOR; Cell Signaling Technology, Danvers, MA, USA, rabbit monoclonal IgG, 1:1000, MW: 289 kDa, catalog no. 5536), Beclin-1 (Cell Signaling Technology, Danvers, MA, USA, rabbit monoclonal IgG, 1:1000, MW: 60 kDa, catalog no. 3495), VPS 34 (Proteintech, Rosemont, IL, USA, rabbit polyclonal IgG, 1:1000, MW: 100 kDa, catalog no. 12452-1-AP), ATG7 (Cell Signaling Technology, Danvers, MA, USA, rabbit monoclonal IgG, 1:1000, MW: 78 kDa, catalog no. 8558), ATG12 (Proteintech, Rosemont, IL, USA, rabbit polyclonal IgG, 1:1000, MW: 48–55 kDa, catalog no. 11264-1-AP), LC3-I/II (MBL International, Woburn, MA, USA, mouse monoclonal IgG, 1:1000, MW: 14 and 18 kDa, catalog no. M152-3), and glyceraldehyde-3-phosphate dehydrogenase (GAPDH; Merck, Darmstadt, Germany, mouse monoclonal IgG, 1:2500, MW: 36 kDa, catalog no. MAB374).
