# Supplementary material for: Estradiol Ameliorates Postmenopausal Bladder Dysfunction by Restoring Mitophagy via the miRNA-200/KLF4/mTOR Signaling Pathway
Source: Int J Mol Sci. 2026 Aug 22;27(17):7529. doi: 10.3390/ijms27177529 (PMC13566216; doi:10.3390/ijms27177529)

Figure 3

Western blot

Protein: ERα

1^0 Ab: Abcam, Rabbit, Monoclonal

Catalog number: ab32063

Molecular weight : 66 kDa

Working concentration: 1:1000

Gel (%): 8%

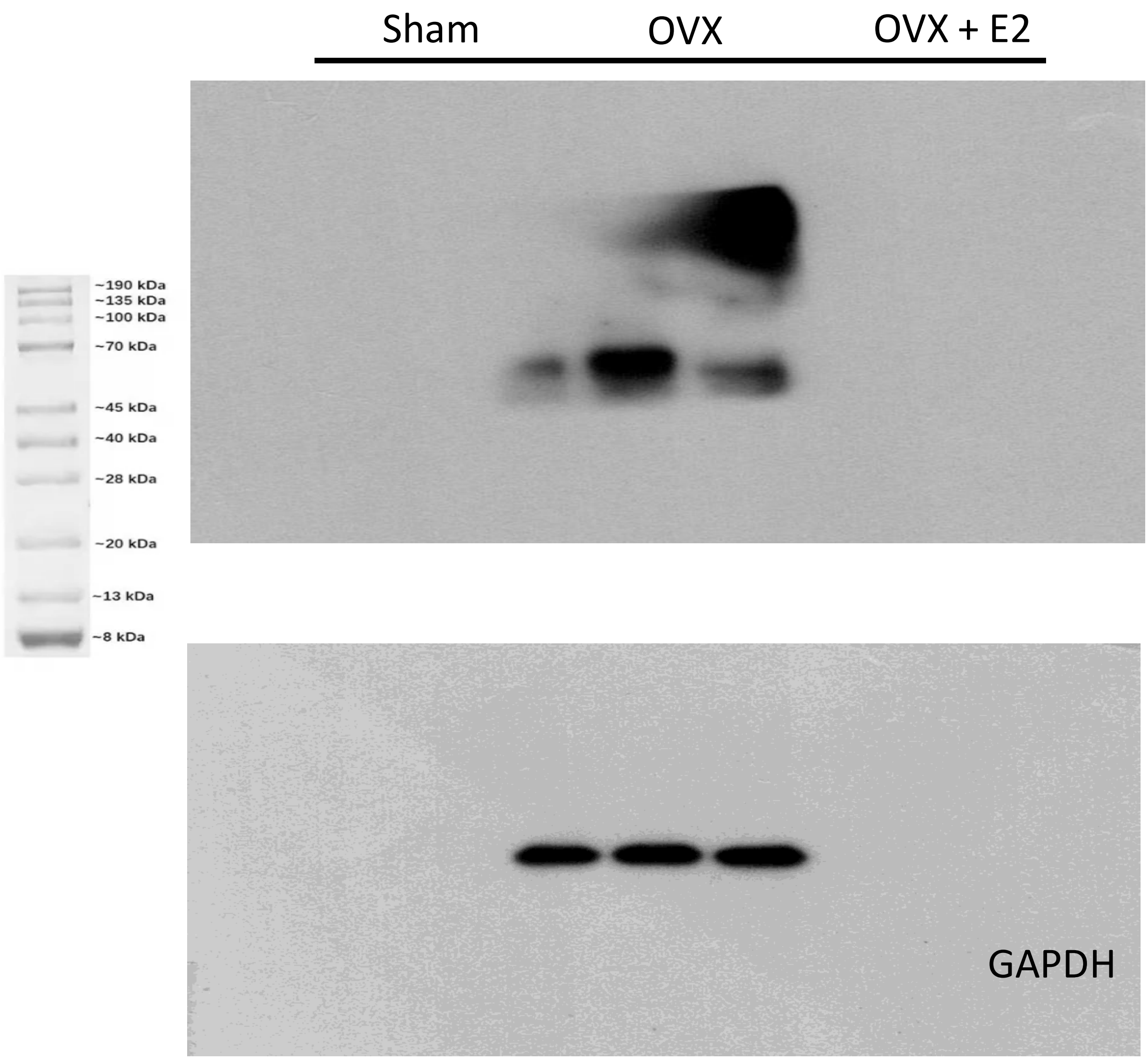

Western blot

Protein: ERβ **1^0 Ab:** Affinity, Rabbit, Polyclonal

Catalog number: #AF6469 Molecular weight : 59 kDa

Working concentration: 1:1000 Gel (%): 8%

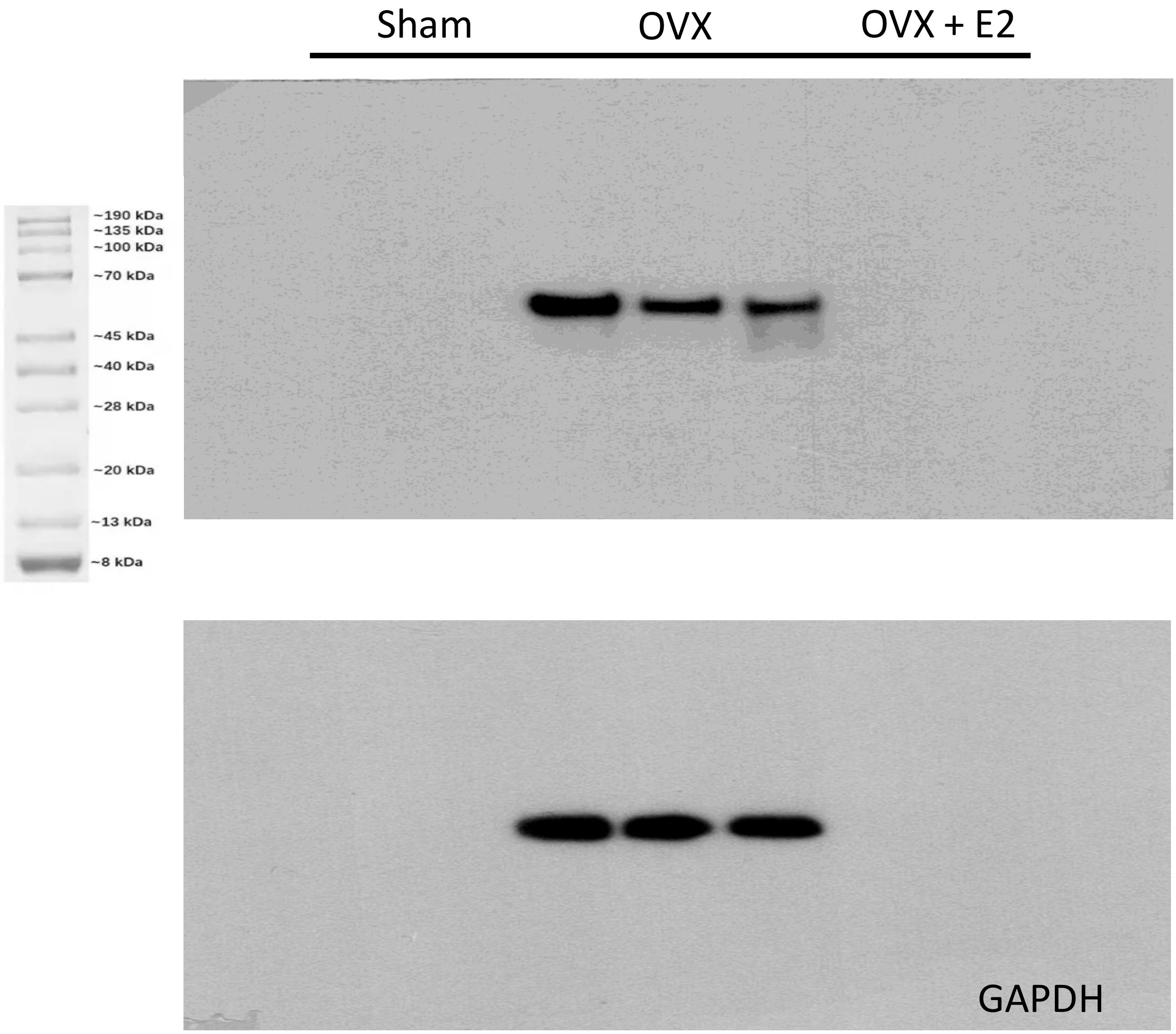

Western blot

Protein: GPER

1^0 Ab: Abcam, Rabbit, Monoclonal

Catalog number: ab260033

Molecular weight : 55 kDa

Working concentration: 1:1000

Gel (%): 8%

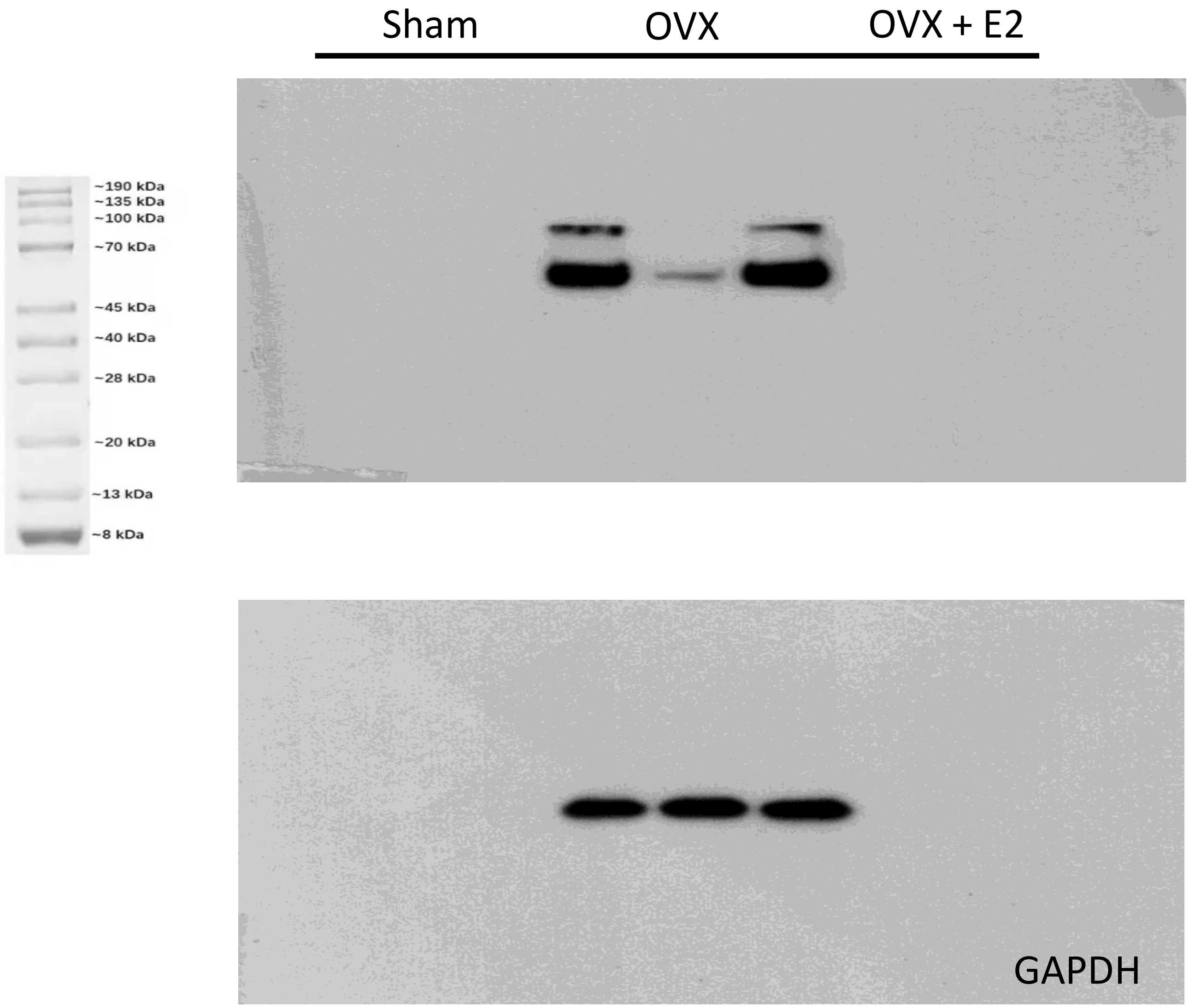

Western blot

Protein: TGF-β1

1^0 Ab: R&D, Mouse, Monoclonal

Catalog number: MAB240

Molecular weight : 50-60 kDa

Working concentration: 1:1000

Gel (%): 8%

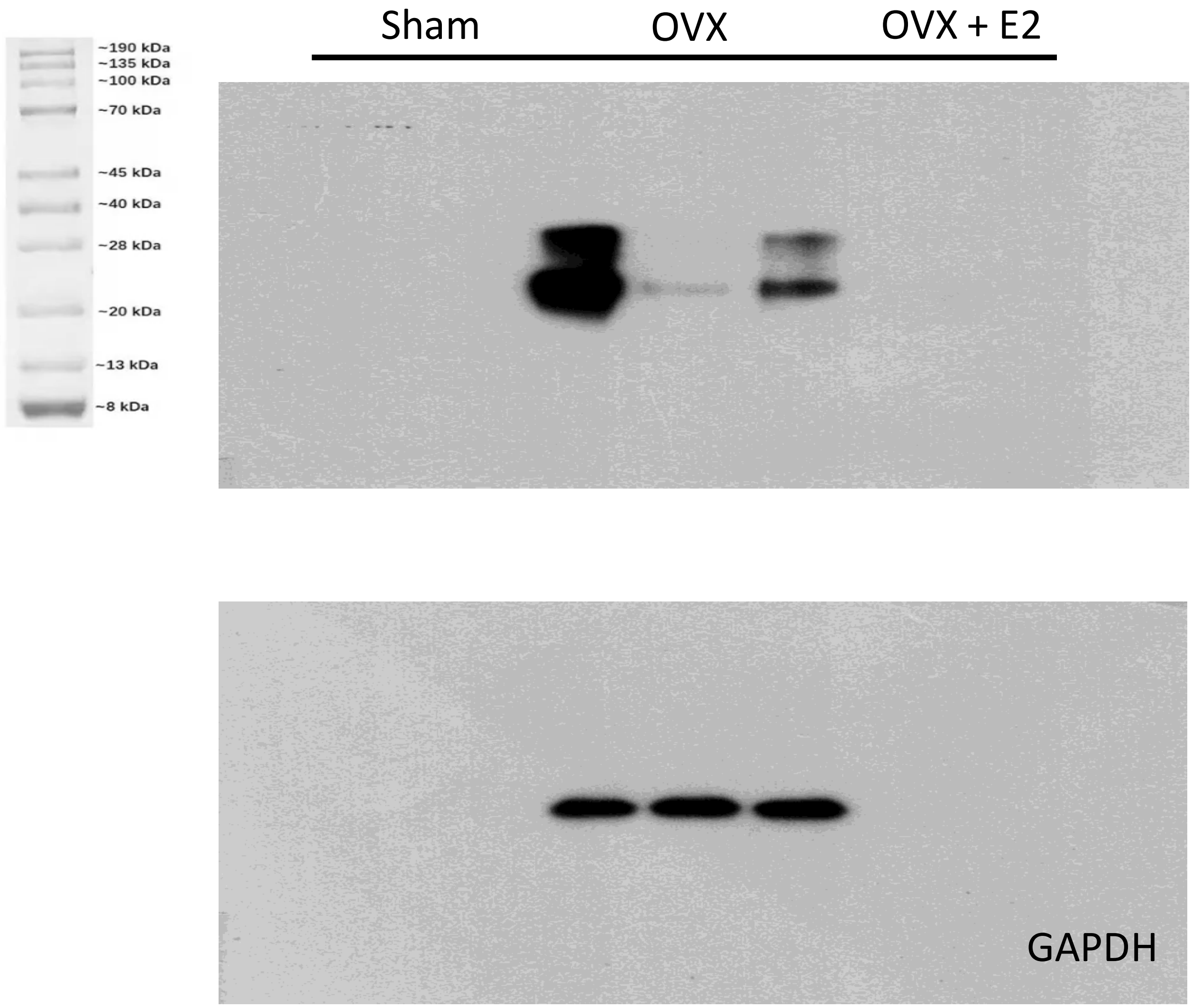

Western blot

Protein: Smad1/5/9

Catalog number: AF0614

Working concentration: 1:1000

1^0 Ab: Affinity, Rabbit, Polyclonal

Molecular weight : 56 kDa

Gel (%): 8%

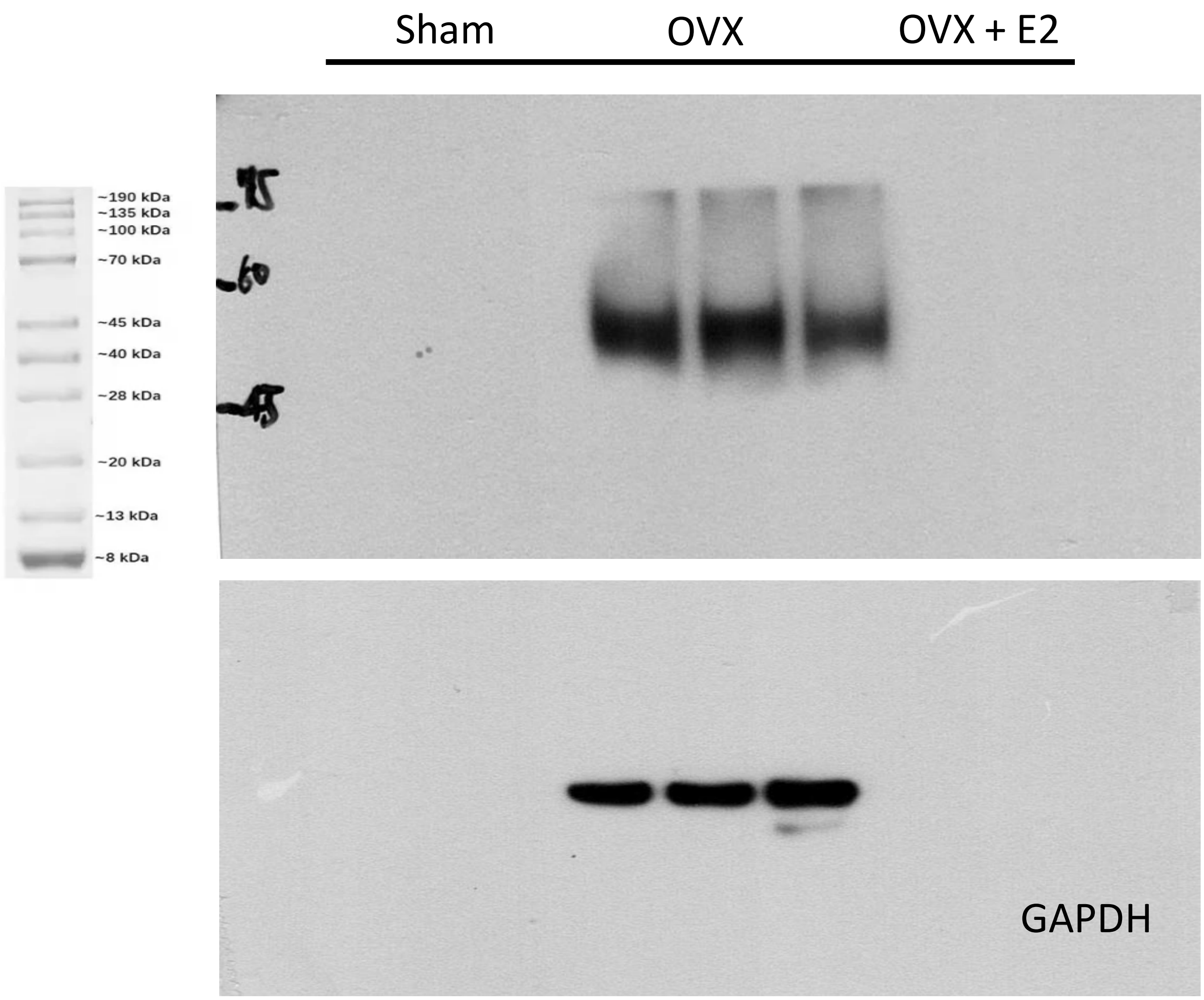

Western blot

Protein: Smad2

Catalog number: 51-1300

Working concentration: 1:1000

1^0 Ab: Thermo, Rabbit, Polyclonal

Molecular weight : 52 kDa

Gel (%): 8%

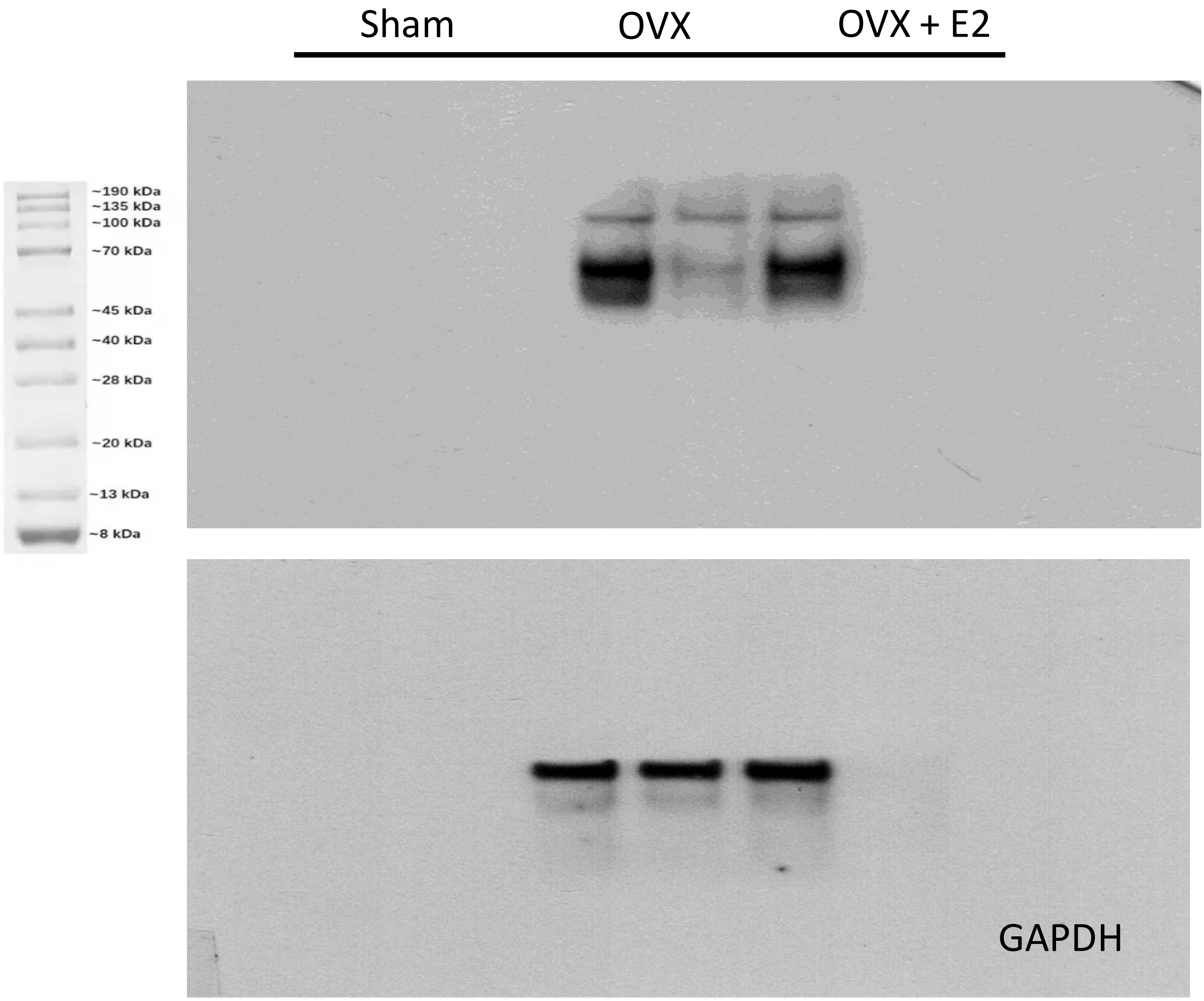

Western blot

Protein: Smad3

Catalog number: PA5-34774

Working concentration: 1:1000

1^0 Ab: Thermo, Rabbit, Polyclonal

Molecular weight : 55 kDa

Gel (%): 8%

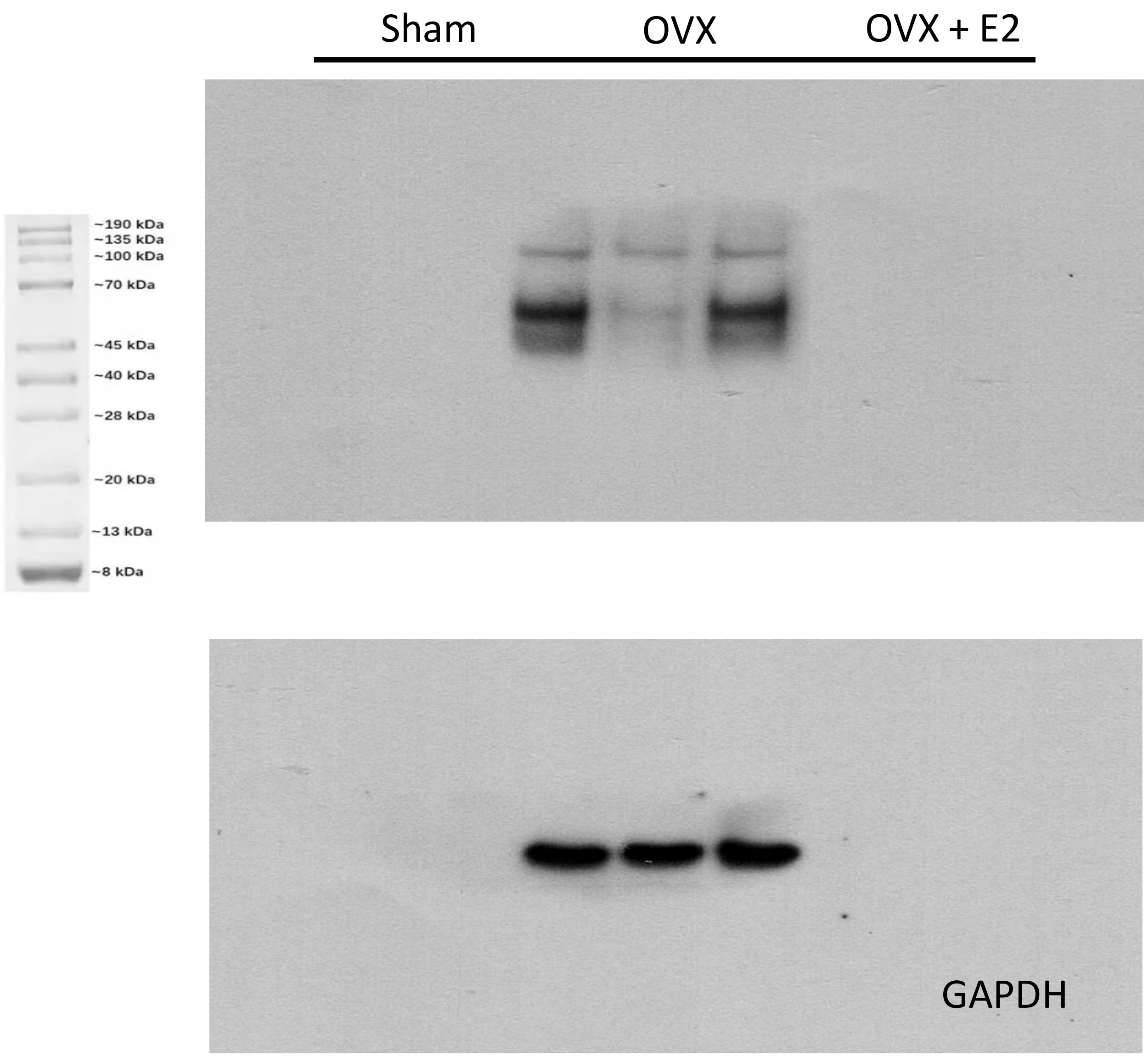

Western blot

Protein: Smad4

Catalog number: MA5-50750

Working concentration: 1:1000

1^0 Ab: Thermo, Rabbit, Polyclonal

Molecular weight : 60 kDa

Gel (%): 8%

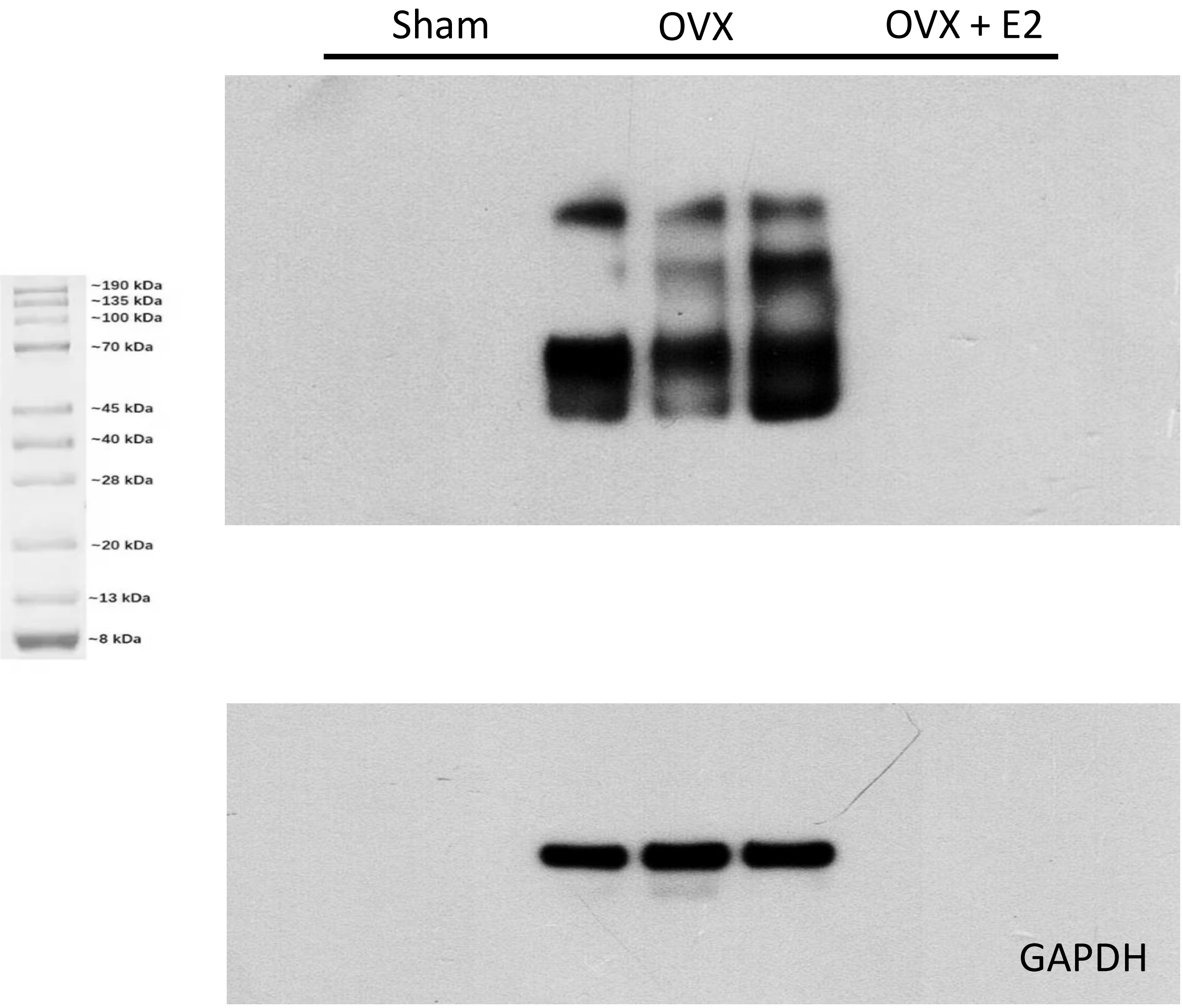

Western blot

Protein: Smad7

1^0 Ab: R&D, Mouse, Monoclonal

Catalog number: MAB2029

Molecular weight : 50 kDa

Working concentration: 1:1000

Gel (%): 8%

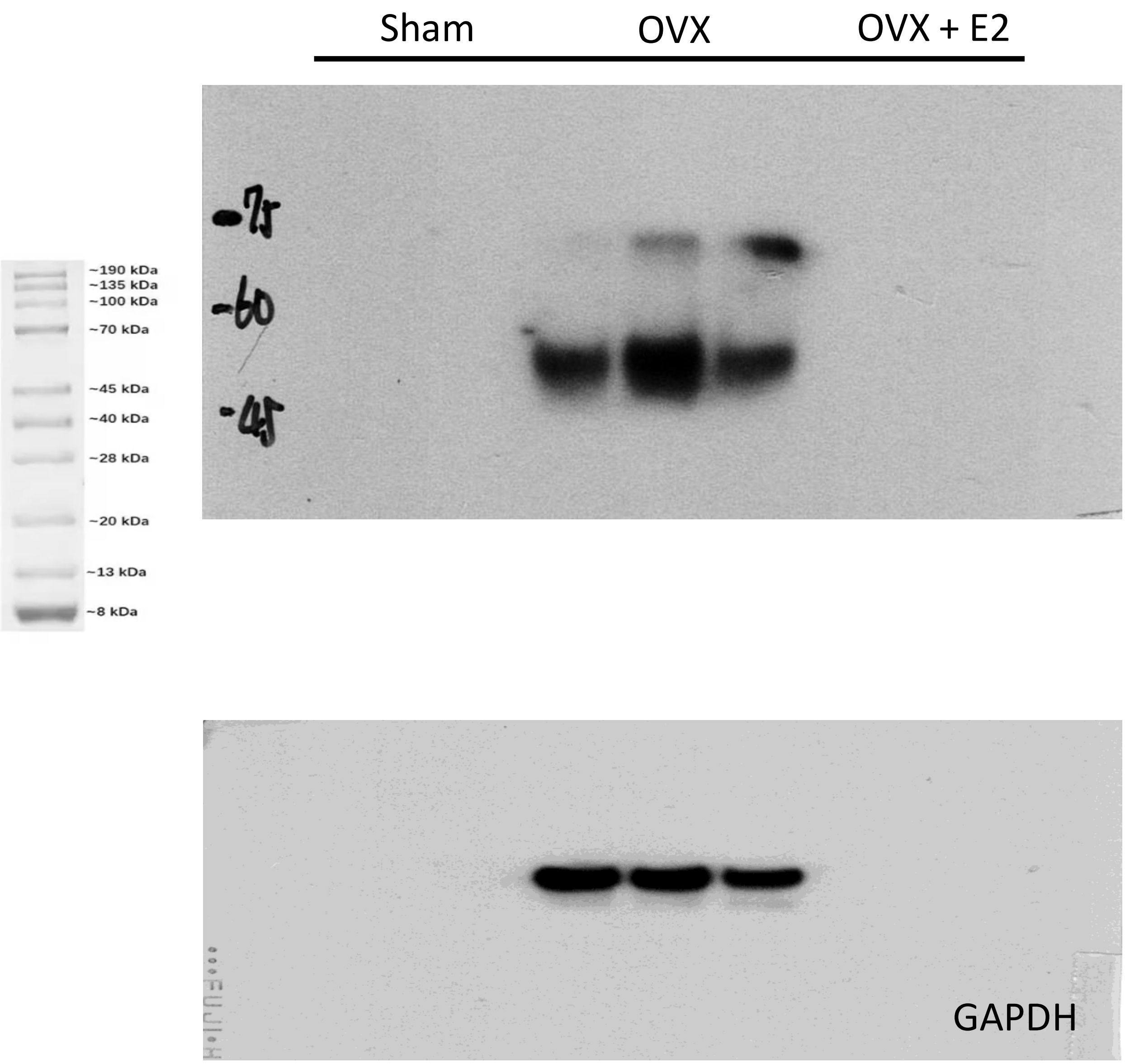

Figure 4

Western blot

Protein: KLF 4

1^0 Ab: Proteintech, Rabbit, Polyclonal

Catalog number: 11880-1-AP

Molecular weight : 50-60 kDa

Working concentration: 1:1000

Gel (%): 8%

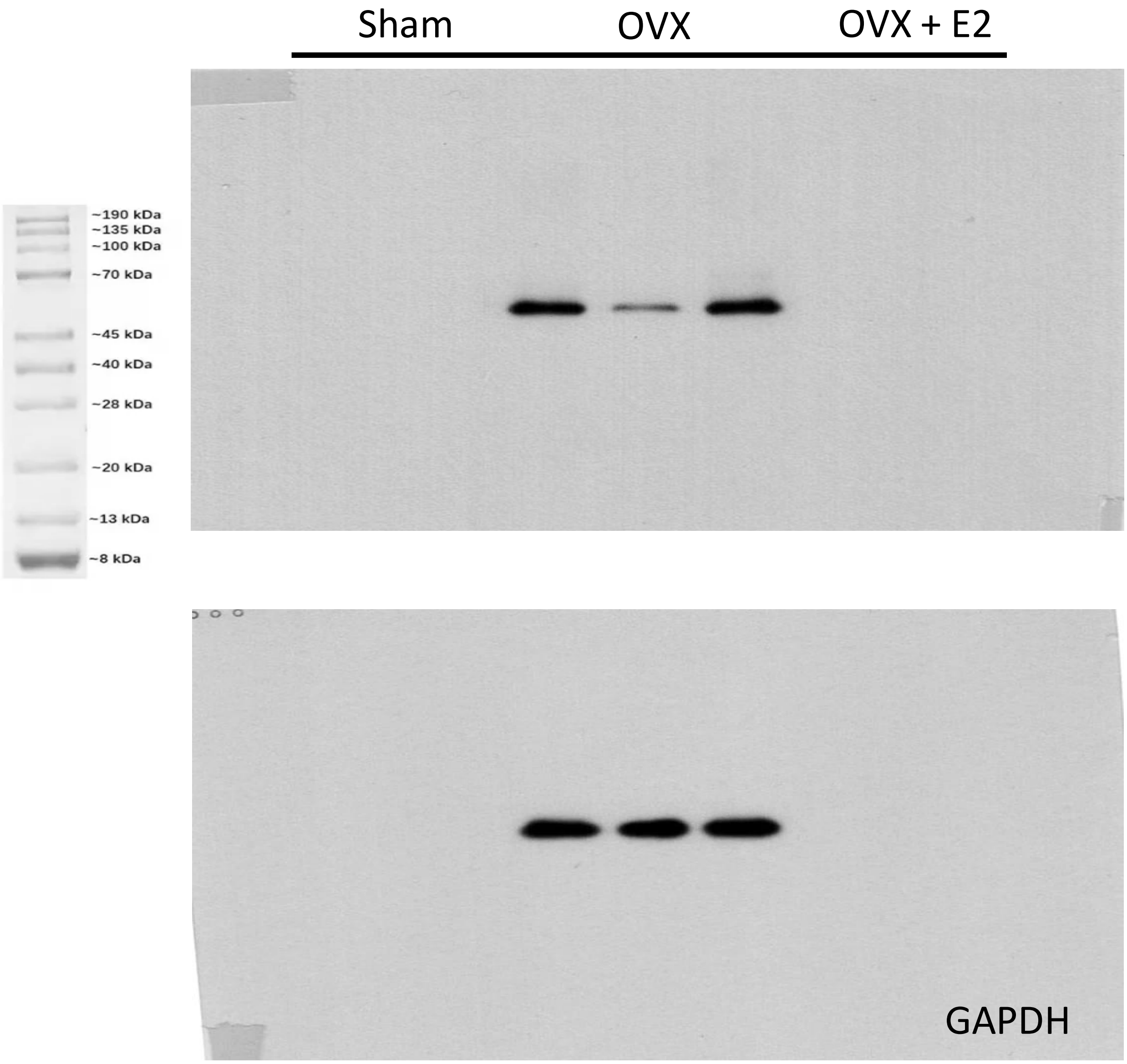

Western blot

Protein: mTOR

1^0 Ab: Cell signaling, Rabbit,monoclonal

Catalog number: 2983

Molecular weight : 289 kDa

Working concentration: 1:1000

Gel (%): 8%

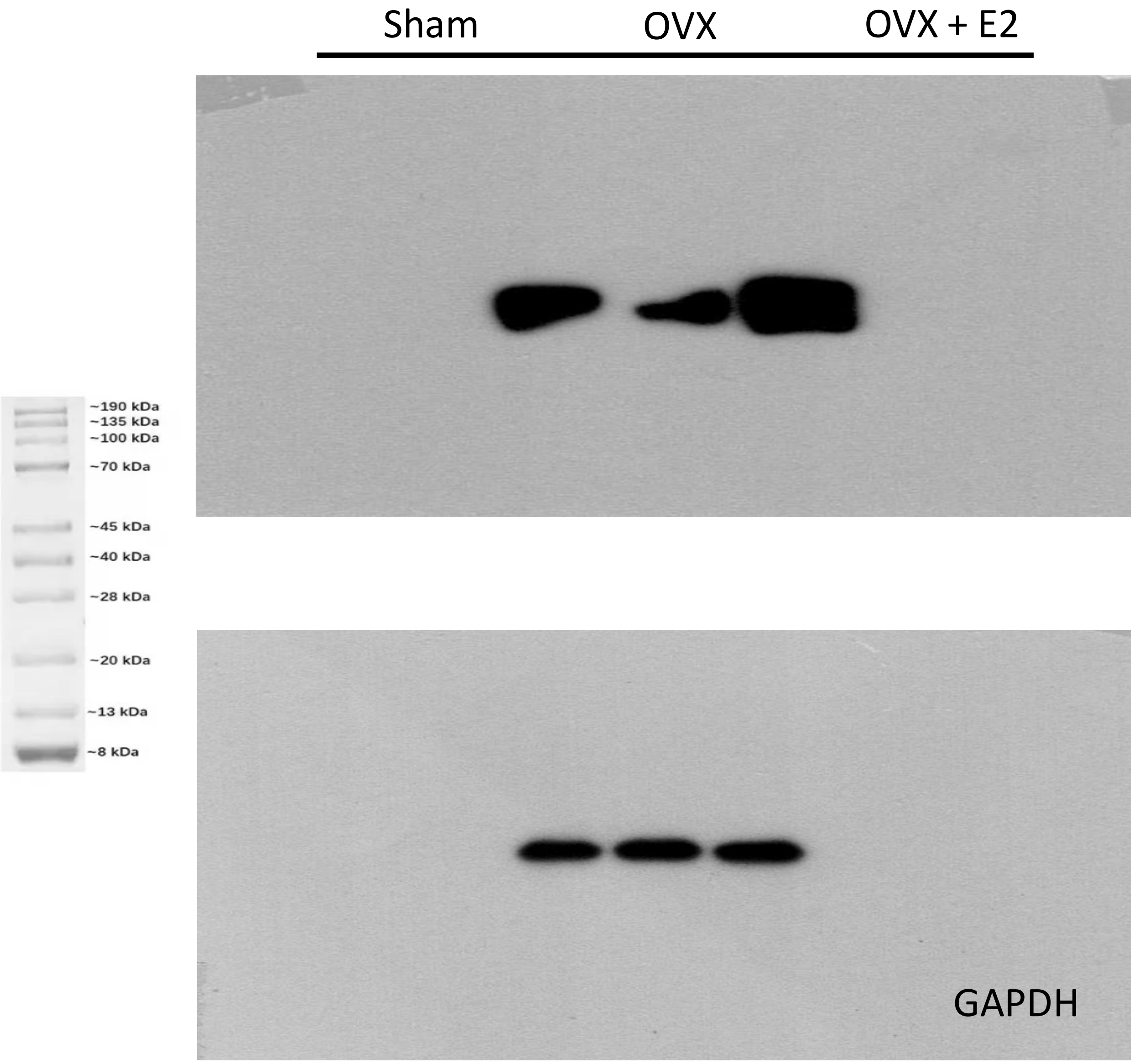

Western blot

Protein: p-mTOR

1^0 Ab: Cell signaling, Rabbit,monoclonal

Catalog number: 5536

Molecular weight : 289 kDa

Working concentration: 1:1000

Gel (%): 8%

Sham	OVX	OVX + E2

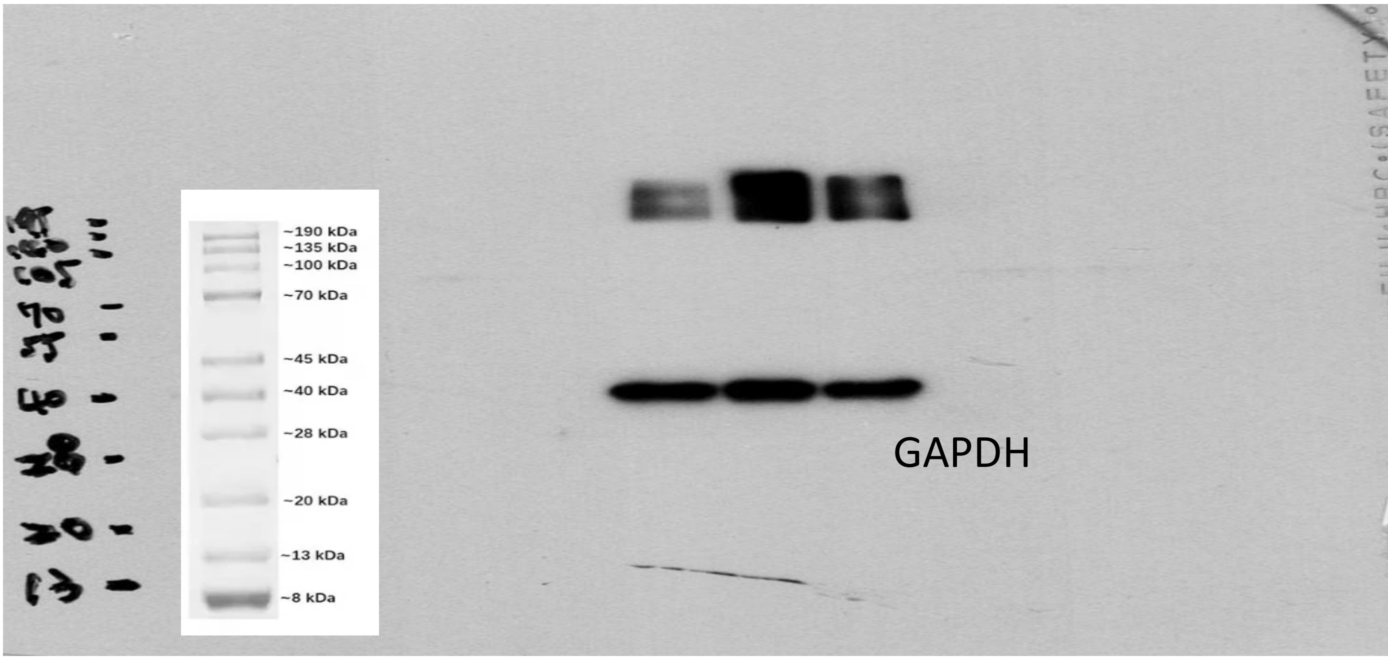

Western blot

Protein: Beclin 1

1^0 Ab: Cell signaling, Rabbit,monoclonal

Catalog number: 3495

Molecular weight : 60 kDa

Working concentration: 1:1000

Gel (%): 8%

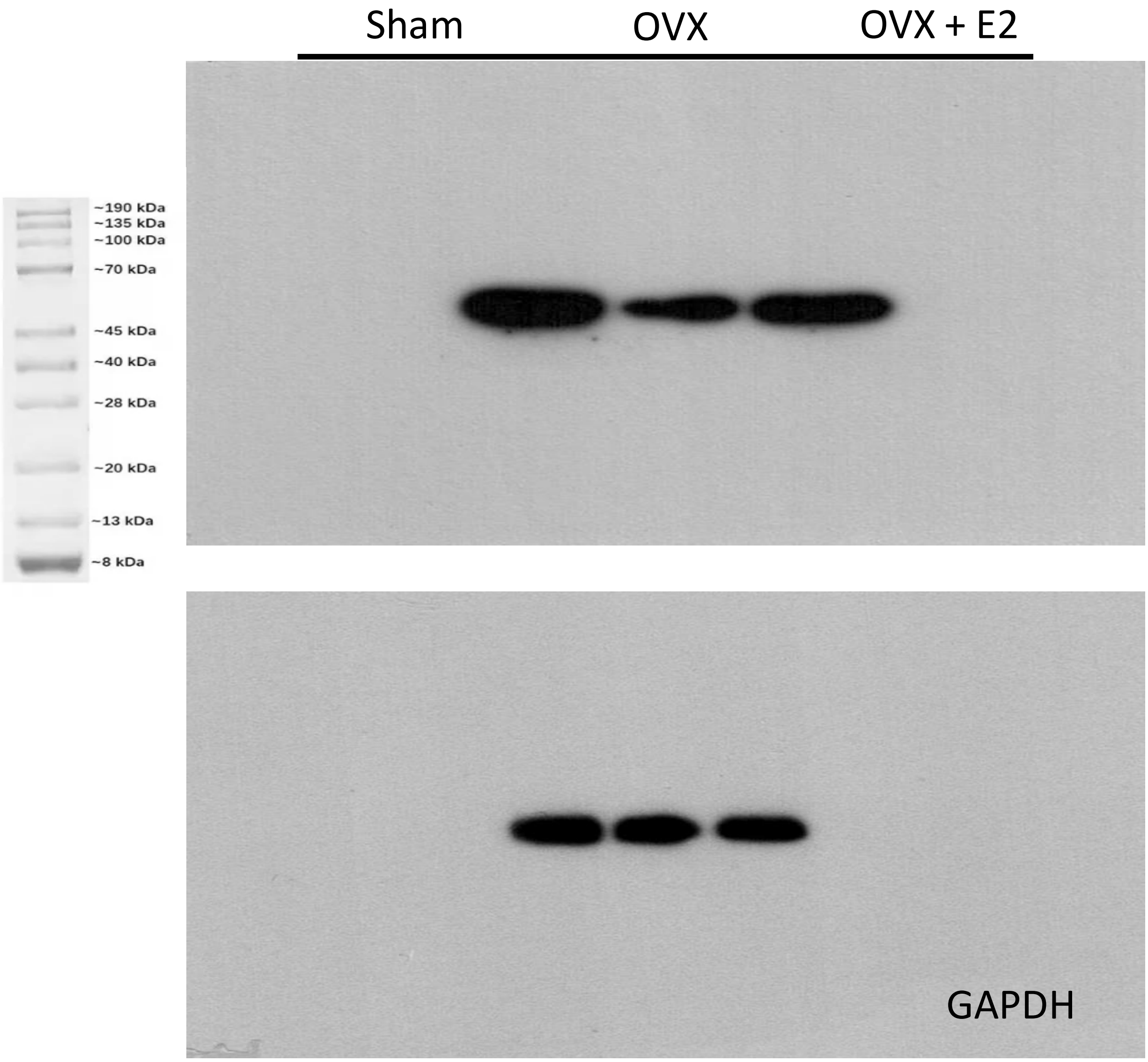

Western blot

Protein: VPS 34 1^0 Ab: Proteintech, Rabbit, Polyclonal

Catalog number: 12452-1-1-AP Molecular weight : 100 kDa

Working concentration: 1:1000 Gel (%): 8%

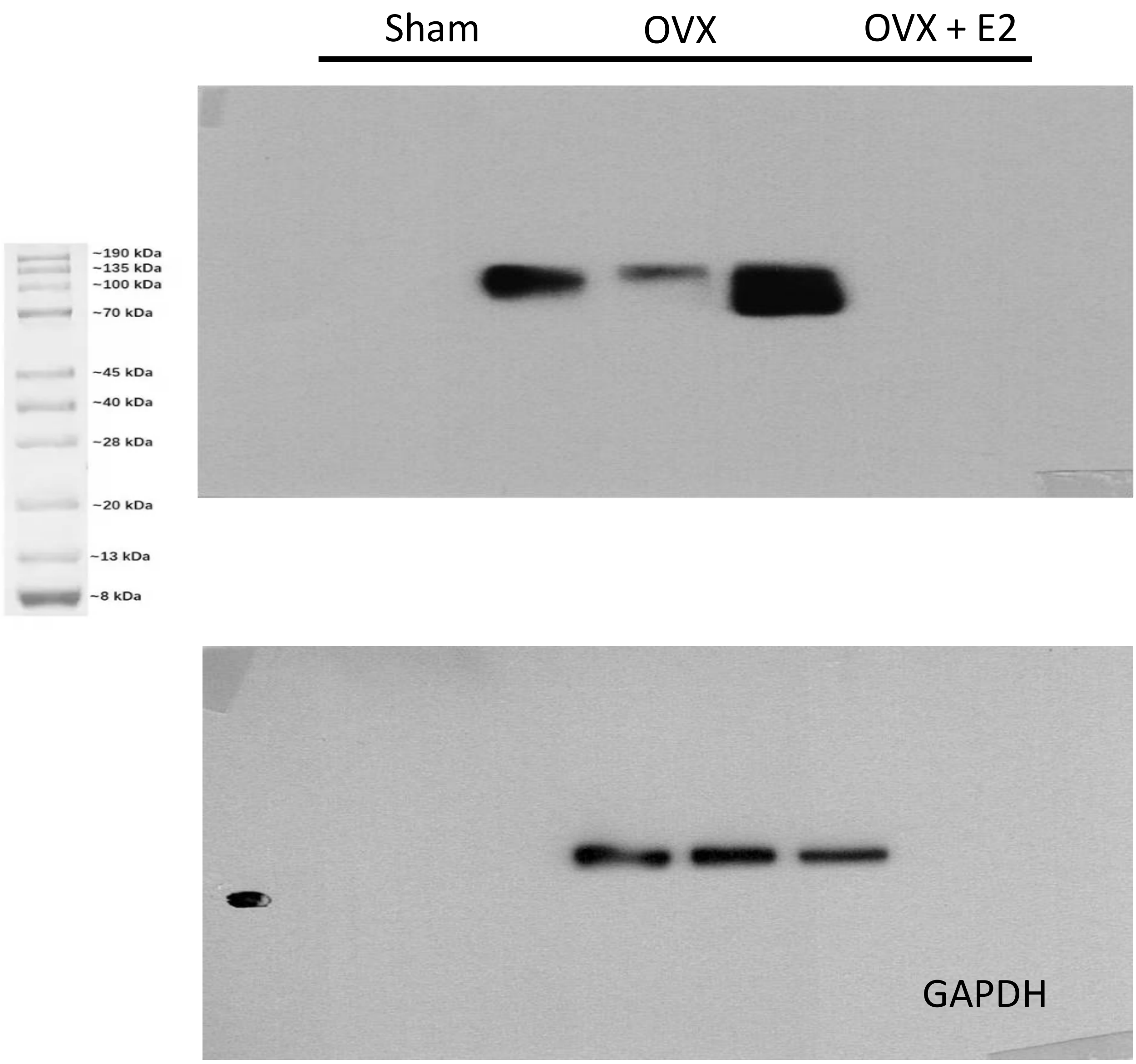

Western blot

Protein: ATG 7

1⁰ Ab: Cell signaling, Rabbit,monoclonal

Catalog number: 8558

Molecular weight : 78 kDa

Working concentration: 1:1000

Gel (%): 8%

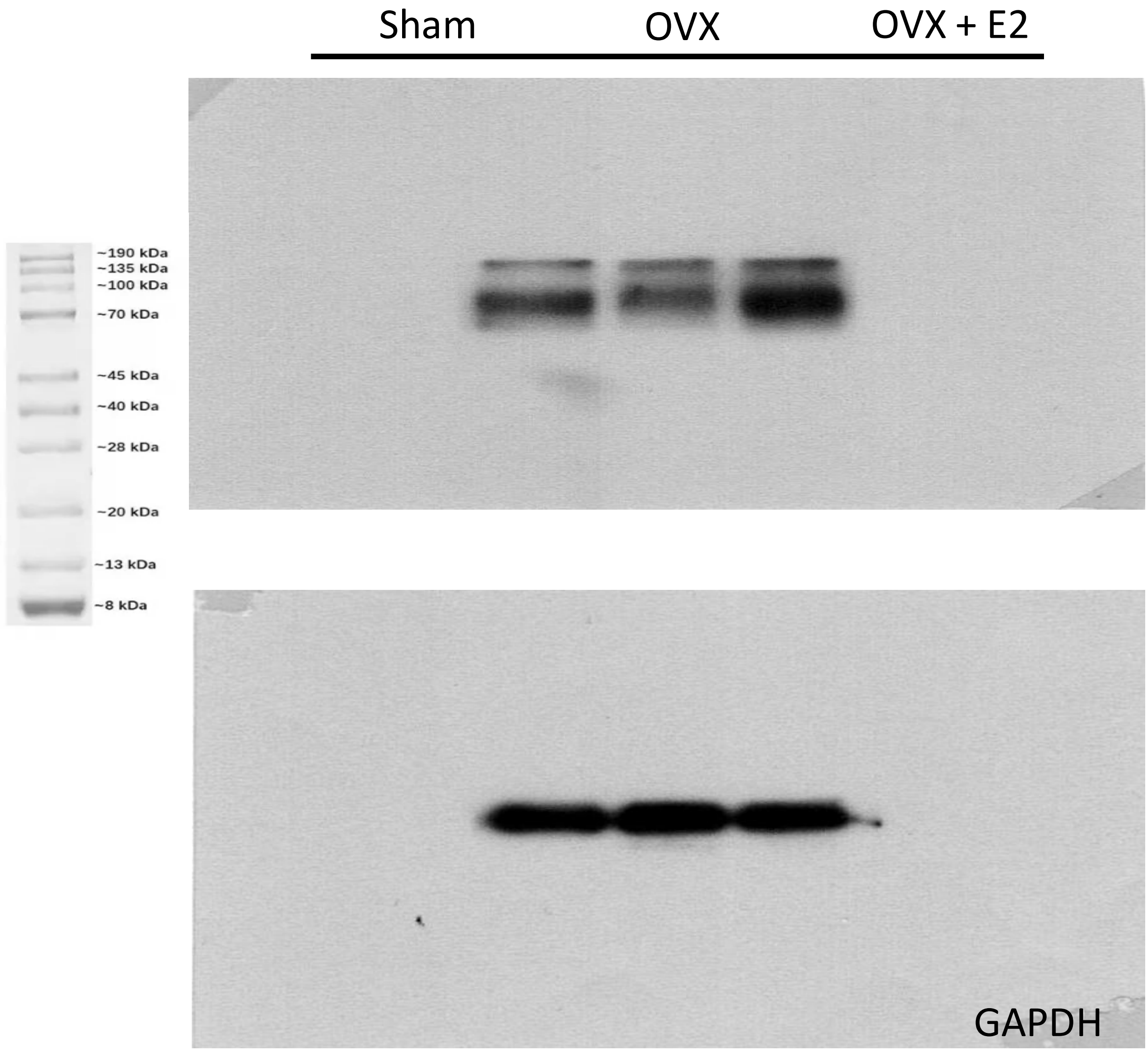

Western blot

Protein: ATG 12

1^0 Ab: Proteintech, Rabbit,Polyclonal

Catalog number: 11264-1-AP

Molecular weight : 55 kDa

Working concentration: 1:1000

Gel (%): 8%

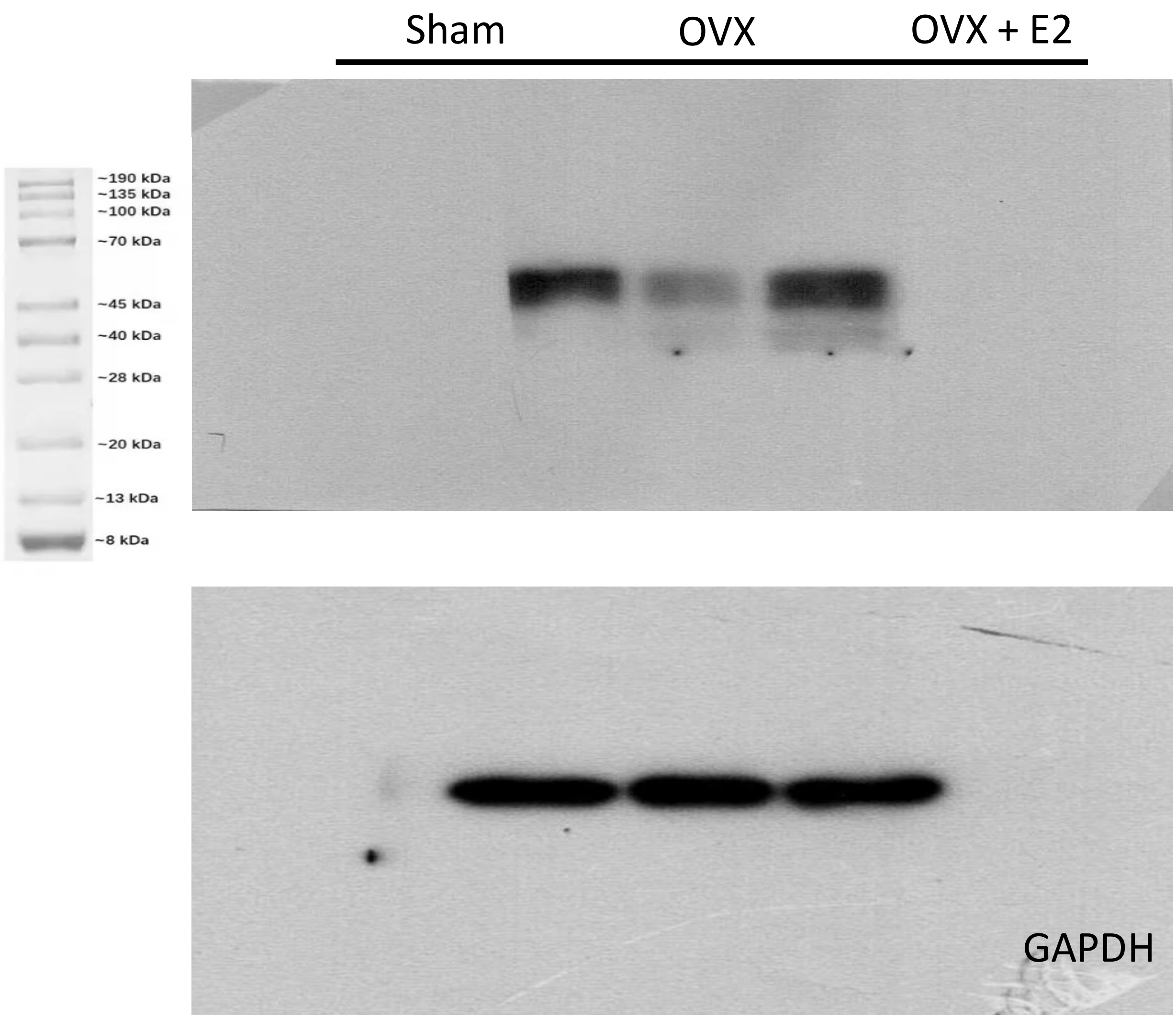

Western blot

Protein: LC3-Ⅰ/Ⅱ

1^{0} Ab: MBL, mouse,monoclonal

Catalog number: M152-3

Molecular weight : 14-16 kDa

Working concentration: 1:1000

Gel (%): 8%

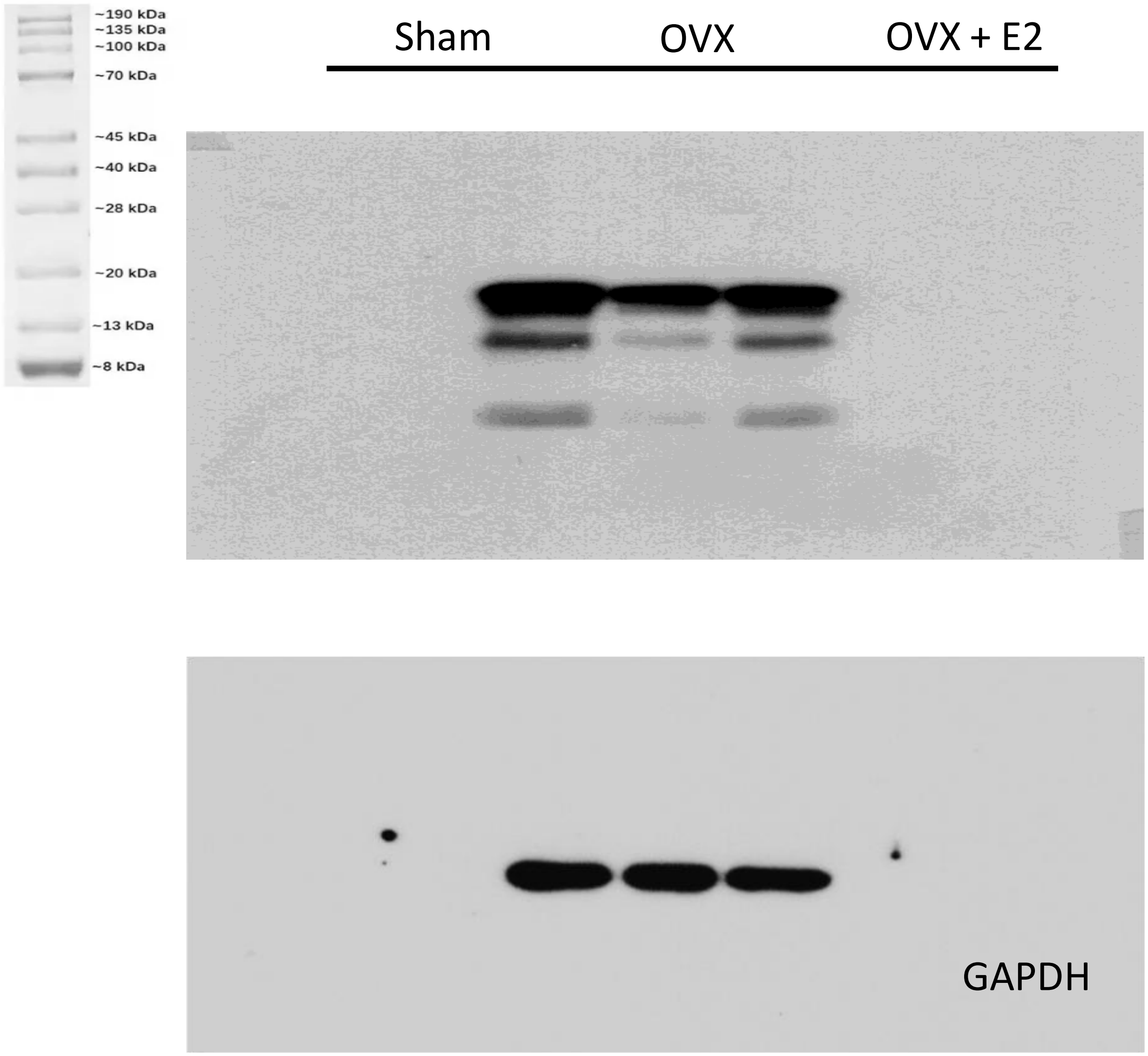

Supplement: Supplementary file 1 [file ijms-27-07529-s001.zip › ijms-4450223-supplementary.pdf]
